# Platelet Rich Plasma and Adipose-Derived Mesenchymal Stem Cells Mitigate Methotrexate-Induced Nephrotoxicity in Rat via Nrf2/Pparγ/HO-1 and NF-Κb/Keap1/Caspase-3 Signaling Pathways: Oxidative Stress and Apoptosis Interplay

**DOI:** 10.3390/toxics11050398

**Published:** 2023-04-22

**Authors:** Farooq A. Wani, Mahrous A. Ibrahim, Shimaa H. Ameen, Amira E. Farage, Zinab Abd-Elhady Ali, Khaldoon Saleh, Medhat M. Farag, Mohammed U. Sayeed, Muhannad A. Y. Alruwaili, Abdulsalam H. F. Alruwaili, Ahmad Z. A. Aljared, Rania A. Galhom

**Affiliations:** 1 Pathology Department, College of Medicine, Jouf University, Sakaka 72388, Saudi Arabia; 2 Forensic Medicine and Clinical Toxicology, College of Medicine, Jouf University, Sakaka 41412, Saudi Arabia; 3 Forensic Medicine and Clinical Toxicology Department, Faculty of Medicine, Suez Canal University (SCU), Ismailia 41522, Egypt; 4 Forensic Medicine and Clinical Toxicology Department, Faculty of Medicine, Zagazig University, Alsharqia 44519, Egypt; 5 Department of Anatomy, Faculty of Medicine, Kafrelsheikh University, Kafrelsheikh 33516, Egypt; 6 Vice Deanship for Academic Affairs, College of Medicine, Imam Abdulrahman Bin Faisal University, Dammam 31441, Saudi Arabia; 7 Medical Biochemistry Department, College of Medicine, Shaqra University, Shaqra 11961, Saudi Arabia; 8 College of Medicine, Jouf University, Sakaka 72388, Saudi Arabia; 9 Human Anatomy and Embryology Department, Faculty of Medicine, Suez Canal University (SCU), Ismailia 41522, Egypt; 10 Center of Excellence in Molecular and Cellular Medicine (CEMCM), Faculty of Medicine, Suez Canal University (SCU), Ismailia 41522, Egypt; 11 Human Anatomy and Embryology Department, Faculty of Medicine, Badr University in Cairo (BUC), Cairo 11829, Egypt

**Keywords:** nephrotoxicity, PCR, methotrexate, caspase-3, MSCs, apoptosis, oxidative stress

## Abstract

Background: the nephrotoxicity of methotrexate (MTX) is observed in high-dose therapy. Moreover, low-dose MTX therapy for rheumatic diseases is debatable and claimed to cause renal impairment. This study aimed at studying the effect of methotrexate in repeated low doses on rat kidneys and assessing the efficacy of adipose-derived mesenchymal stem cells (AD-MSCs) and platelet rich plasma (PRP) for attenuating this effect. Methods: Forty-two male Wistar rats were used, 10 rats were donors of AD-MSCs and PRP, 8 rats served as control, and the remaining rats were subjected to induction of nephrotoxicity by MTX intraperitoneal injection once weekly for successive 8 weeks and then assigned into 3 groups of 8 animals each: Group II: received MTX only. Group III: received MTX + PRP. Group IV: received MTX + AD-MSCs. After one month, rats were anaesthetized, serum-sampled, and renal tissue removed for biochemical, histological, and ultrastructural evaluation. Results: there was significant tubular degeneration, glomerulosclerosis, fibrosis, decreased renal index, along with increased levels of urea and creatinine in the MTX group compared to the control group. Immunohistochemical expression of caspase-3 and iNOS in the renal tissue was significantly increased in group II compared to groups III and IV. Biochemical results revealed higher tissue malondialdehyde (MDA) concentration in the MTX-injected group which decreased significantly in co-treatment with either AD-MSC or PRP + MTX. MSC promoted the activation of the Nrf2/PPARγ/HO-1 and NF-κB/Keap1/caspase-3 pathways, increased antioxidant enzyme activities, reduced lipid peroxidation levels, and alleviated oxidative damage and apoptosis. PRP showed therapeutic effects and molecular mechanisms similar to MSC. Furthermore, MSC and PRP treatment significantly reduced MTX-induced upregulation of the pro-inflammatory (NF-κB, interleukin-1ß, and TNF-α), oxidative stress (Nrf-2, hemoxygenase-1, glutathione, and malondialdehyde), and nitrosative stress (iNOS) markers in the kidney. Conclusion: repeated administration of low-dose MTX resulted in massive renal tissue toxicity and deterioration of renal function in rats which proved to be attenuated by PRP and AD-MSCs through their anti-inflammatory, anti-apoptotic and anti-fibrotic properties.

## 1. Introduction

Methotrexate (MTX) is a commonly used folate antagonist which primarily inhibits DNA synthesis. It has high efficacy in the management of rheumatoid arthritis and many other inflammatory and rheumatic diseases [1,2]. Even with the low-dose therapy in such diseases, serious adverse effects have been attributed to MTX therapy, for example, myelosuppression, infection, hepatotoxicity, and lung diseases [3,4,5]. Recent studies determined that the risk of nephrotoxicity with low-dose MTX is higher than previously thought and should be put under consideration [6,7]. 

Methotrexate (MTX), being an antimetabolite drug is used extensively to treat cancers and autoimmune diseases [8]. High-dose methotrexate (HDMTX) followed by leucovorin rescue is successfully used to manage childhood and adult neoplasms. HDMTX is securely delivered to patients having normal renal function along with proper hydrating and alkalizing strategies [9].

HDMTX is defined as a dose higher than 500 mg/m^2^, especially used to treat ALL, lymphomas, and osteosarcoma [10,11]. Although HDMTX is well tolerated but it can cause considerable toxicity including acute kidney injury (AKI) in patients with impaired renal function, dehydration, acidic urine, and drug interactions. The toxicity results from the precipitation and crystallization of methotrexate and its metabolites in the tubular lumen, resulting in tubular necrosis [12]. Despite the best adjunctive treatment, AKI occurs in 2–12% of patients [10]. AKI becomes a harbinger for other life-threatening toxicities including myelosuppression and mucositis [9].

MTX exerts its toxicity in non-cancerous cells by triggering oxidative stress and impairs the mitochondrial activity by building up reactive oxygen species (ROS) [10]. Oxidative stress could be prompted in MTX toxicity by suppressing the nuclear factor (erythroid derived 2)-like 2 (Nrf2), which is a transcription factor. The detoxification response and antioxidant regulation as well as the homeostasis of the cellular redox are adjusted by Nrf2 [13].

Platelet rich plasma (PRP) is a biological product defined as a portion of the plasma fraction of autologous blood with a platelet concentration above the baseline (before centrifugation) [14]. PRP is rich in platelets, growth factors, clotting factors, cytokines, and chemokines [15,16]. The important growth factors and cytokines isolated from PRP include VEGF, TGFβ, PDGF, EGF, IGF, platelet factor 4, IL-1, fibronectin, osteocalcin, osteonectin, thrombospondin-1, etc. [17,18]. PRP has been found to facilitate the healing process and has been used in musculoskeletal injuries including tendonitis, rotator cuff injuries, tennis elbow, Achilles tendon injuries, muscle injuries, and bone repair [16]. The use of PRP in oral and maxillofacial surgeries such as implants, bone grafts, and reconstructive surgeries has been associated with healing rates which are 2–3 times that of the normal surgical sites and improved surgical results [19]. The use of PRP in dermatology has seen an increasing trend especially for wound healing, treatment of alopecia, tissue regeneration, scar revision, and fat grafting [16]. PRP has shown promising results in animal studies of nephrotoxic models, whereas it has been found to induce the regeneration of epithelial cells, suppression the inflammation, and reduction in tubulointerstitial fibrosis [20,21].

Mesenchymal stem cells (MSCs) isolated from a variety of adult tissues have the capability to differentiate into multiple cell lineages [22,23]. MSCs have immunomodulatory, anti-inflammatory, anti-proliferative, reparative, and regenerative effects [22,24]. The regenerative capacity is due to paracrine effects mediated by the secretion of cytokines, growth factors, microvesicles, and exosomes for cell-to-cell communication with the injured renal cells [25]. 

In animal models, MSCs possess reparative actions in AKI through increased SIRT3 activity, preserving mitochondrial homeostasis and organelle trafficking [25]. MSC treatment causes decreased expression of IL-6, IL-1b, and TNF-α and increased expression of IL-4 and IL-10 [26]. In a rat model, MSC treatment ameliorated the nephrotoxic and hepatotoxic effects of MTX by decreasing oxidative stress and reducing expression of NF-κB and caspase-3 [27]. Keeping in view the potential role of MSCs in AKI and CKD animal models, we planned the study to evaluate protective effects of AD-MSCs and PRP against nephrotoxicity induced by methotrexate by investigating the signaling pathways’ role of Nrf2/PPARγ/HO-1 and NF-κB/Keap1/caspase-3 in the inducement and abatement of renal damage.

## 2. Materials and Methods

The laboratory and experimental work of this research were performed in the tissue culture lab and animal house of the Center of Excellence of Molecular and Cellular medicine (CEMCM), Faculty of Medicine, Suez Canal University (FOM-SCU). The electron microscopic examination was carried out in Mycology and Biotechnology Center, Al-Azhar University (Cairo, Egypt). All experimental procedures and animal maintenance were conducted in accordance with the recommendations in the Guide for the Care and Use of Laboratory Animals of the National Institutes of Health. The rats were housed in controlled temperature and humidity. They were kept in separate cages with allowance for water and rodent food. 

### 2.1. Methotrexate

Methotrexate was obtained from Baxter Company (Cairo, Egypt). Diagnostic and research reagents, chemicals, and solvents were purchased from a Bio-diagnostic company for diagnostic and research reagents (Dokki, Giza, Egypt) and Sigma-Aldrich Chemical Co., Louis, Missouri, USA. All other chemicals, reagents and solvents were of the highest purity and analytical grade available from Sigma (St. Louis, MO, USA). Drug solutions were freshly prepared before animal treatments.

### 2.2. Experimental Design

**Scheme 1:**
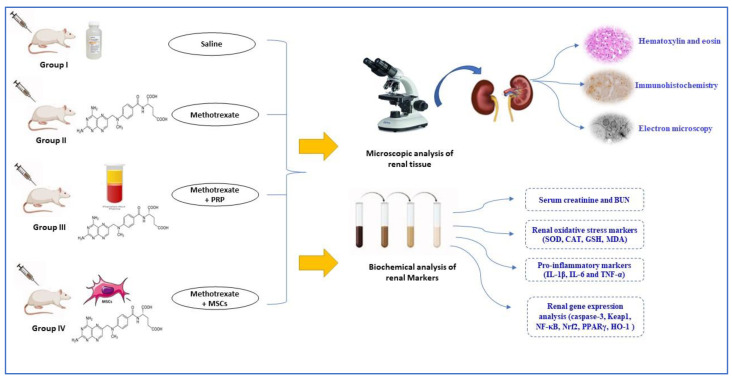
A schematic diagram representing the study design.

Forty-two adult Wistar male rats (weighing 180–220 g each and aged 3–4 months) were used in the current study. They were allowed 1 week of adaptation prior to experimental study and were given free access to rodent diet and water in 20–25 °C and 60–70% humidity. Of these rats, 10 of them were used as a donor for adipose-derived mesenchymal stem cells (AD-MSCs) and protein rich plasma (PRP), and the remaining animals were assigned into 4 groups containing 8 rats each. All the study groups were as following (Scheme 1):

Group I control-group rats received PBS equivalent to the amount of other injections.
Group II (MTX): induction of renal toxicity was performed by MTX (1 mg/kg) injected intraperitoneally in each rat once a week for 8 consecutive weeks [28,29].
Group III (MTX + PRP): the animals which were subjected to MTX-induced nephrotoxicity (as group II) were treated with PRP in a dose of 0.5 mL/kg. Each animal received 3 doses subcutaneously with 2 days interval starting 2 days after the last dose of MTX [30,31].
Group IV (MTX + MSC): the animals were subjected to MTX-induced nephrotoxicity (as group II) and received 3 × 10^6^ AD-MSCs 2 days after the last dose of MTX and repeated 6 days after.

### 2.3. Induction of MTX Nephrotoxicity

The dose of MTX was administered to rats of group II, III, and IV once weekly for 8 successive weeks in a dose of 1 mg/kg through an intraperitoneal route [28,29].

### 2.4. Isolation of AD-MSCs

Omental fat of donor rats were excised under strict aseptic condition, washed several times with phosphate buffered saline (PBS) supplemented with 3% penicillin/streptomycin (P/P) (Lonza). Adipose tissue samples of 0.5–1 g were placed in a 15 mL tube and washed 3 times with equal or double volume of PBS, cut into fine fragments, and incubated with 0.1% collagenase type I for enzymatic dissociation of cells and obtaining stromal vascular fraction (SVF). Incubation with collagenase took place for 60 min in a 37 °C shaking water bath at 250 rpm. SVF was passed through a cell strainer (100 μm) and centrifuged at 1800 rpm for 10 min. After discarding the supernatant, the cell pellet was resuspended in PBS, passed through a cell strainer (70 μm), and centrifuged at 1800 rpm for 10 min. The resulting pellet was re-suspended in complete media (DMEM supplemented with 10% FBS and 1% p/p solution), seeded in Petri dishes, and monitored on daily basis with frequent media exchange (every 3–5 days) until reaching confluency. Trypsinization of primary culture cells (passage 0, P0) was performed around the 10th day of culture. The cells were expanded through P1, 2, and 3, then trypsinization was conducted for characterization and transplantation [32].

### 2.5. Flow Cytometric Phenotypic Analysis of the Isolated Cells

Characterization of the isolated AD-MSCs was dependent on their morphological criteria (fibroblast-like appearance during phase contrast examination of all passages and colony formation), physical property (plastic adherence), and specific surface antigen expression. A flow cytometric analysis was carried out in the Oncology Diagnostic Unit, FOM-SCU. The cells were analyzed in the 3rd passage (P3), an aliquot of 1 × 10^4^ cells was evaluated using flow cytometry for surface markers of mesenchymal stem cells (CD105) and hematopoietic stem cells markers (CD45, and CD34) (all from Santa Cruz Biotechnology, Dallas, TX, USA). Cells were incubated in staining buffer (PBS with 2% FBS and 0.1% sodium azide) containing anti-CD105, anti-CD45, and anti-CD34 for 30 min on ice. Cells stained with the appropriate isotype-matched immunoglobins were used as negative controls. After staining, the cells were fixed with 2% *w/v* paraformaldehyde and analyzed by NAVIOS flow cytometer (Beckman Coulter, Miami, FL, USA) [33].

### 2.6. Preparation of PRP

The PRP preparation was carried out by adapting the protocol of the double centrifugation tube method [30,34]. Rats were anesthetized with ether, the whole animal blood was collected under aseptic technique through cardiac puncture 3.2% sodium citrate, then gathered into tubes with 0.3 mL of the anticoagulant (Omental fat was obtained from the same animals for isolation of AD-MSCs).

The blood was centrifugated twice. For 10 min and at 1600 revolutions per minute (rpm), the tubes were centrifuged in the first centrifugation. Three different density compartments resulted from this centrifugation: red blood cells in the inferior layer, buffy coat of white blood cells in the intermediate layer, and plasma in the superior layer. Without disturbance of the buffy coat, the plasma was pipetted, and the fraction just above buffy coat was gained. For 10 min and at 2000 rpm, the plasma was centrifuged again. Two portions resulted from this centrifugation: the platelet button at the bottom and platelet-poor plasma (PPP) at the top. A portion of PPP was discarded, and a portion was retained with platelet button in the tube which was then gently pipetted to enhance platelets resuspension. Platelet-rich plasma (PRP) is produced from the previous procedure. The concentration of platelet was confirmed by counting a sample of 80 μL of the PRP in an automatic apparatus to confirm that platelet count was more than 1,000,000/μL. Then, PRP was allocated and frozen at −80 °C for use.

### 2.7. Administration of PRP and AD-MSCs Transplantation

In the administration of PRP, phosphate buffer saline (PBS) was used to dissolve 0.5 mL/kg PRP (PRP 1:1 PBS), then it was aspirated immediately with a micropipette, placed in a sterile insulin syringe, and injected subcutaneously in each rat of group III 2 days after the last dose of MTX and repeated twice more with 2 days interval [30,31]. The cultured AD-MSCs of P3 were trypsinized, tested for viability, counted, and allocated. Each animal in group IV received 3 ×10^6^ cells, resuspended in 0.5 mL PBS, intravenously in the tail vein 2 days after the last dose of MTX and received the same dose 6 days after [35].

### 2.8. Acquisition of Specimen and Histopathological Examination

One month after the last dose of PRP and AD-MSCs, all animals of all groups were euthanized. Renal tissues were fixed in 10% formalin and embedded in paraffin blocks. Tissue sections were cut at 4 μm thickness, deparaffinized, and stained with hematoxylin and eosin (H&E), Masson trichrome, and periodic acid Schiff (PAS). A semiquantitative renal scale (histopathological score) was used to grade and evaluate the degree of tubular changes in H&E-stained sections (including vacuolization, hyaline casts, tubular epithelium degeneration, intertubular hemorrhage, dilated tubular lumen, and necrosis). These parameters were evaluated under a 4-point scale: (−)  =  no change, (+)  =  10–25% mildly affected tubules, (++)  =  25 to 50% moderately affected tubules, and (+++)  =  more than 50% severely affected tubules. For this purpose, 10 non-overlapping fields for each rat in all groups were examined blindly under a light microscope with high magnification (×400) [30]. 

### 2.9. Immunohistochemical Staining

For immunohistochemical expression of caspase-3 (Casp-3) and inducible nitric oxide (iNOS) in the kidney tissue, 4 µm thick paraffin sections were deparaffinized, followed by antigen retrieval and incubation at 4 °C with the primary antibody against Casp-3 and iNOS (Abcam, Cambridge, UK) overnight. Then, sections were incubated with peroxidase-conjugated secondary antibody. Sections were then counterstained with Mayer’s hematoxylin. The immunoreaction for Casp-3 and iNOS was visualized using a light microscope [36]. 

### 2.10. Morphometric Examination of Renal Sections

Image J software (1.49 v/Java 1.6.0_244 (64-bit)” (National Institutes of Health, Bethesda, MD, USA) was used to assess the following parameter in renal tissues:

1. Mesangial index in renal cortex stained with PAS X 400. The mesangial matrix area was defined as the PAS–positive area within the tuft area. So, it could be calculated as the ratio of mesangial matrix area divided by the tuft area [29].
2. Relative glomerular and interstitial fibrosis area in Masson-stained sections X 400 [36].
3. Optical density of Casp-3 and iNOS immunohistochemically stained sections. After subtracting the background noise, six non overlapping fields from each sample were used to calculate the average positive Casp-3 and iNOS immunoactivity [37].

### 2.11. Transmission Electron Microscopy

For the ultrastructure examination of renal tissue, tissue preparation was performed as follows: Renal cortical specimens were immersed in 2.5% glutaraldehyde in 0.1 mol/L cacodylate buffer (pH 7.2, 2 h, 4 °C), postfixed in 0.1 mol/L osmium tetroxide (2 h, 4 °C), and then embedded in Epon following dehydration in a graded series of ethanol preparations. Semithin sections (1 μm thick) were cut, stained with Toluidine blue, examined, ultra-thin sections were stained with uranyl acetate and lead citrate, and then examined and photographed with TEM 10 transmission electron microscope (Zeiss, Jena, Germany) in the regional center for mycology and biotechnology, electron microscopy unit, Al-Azhar University (Cairo, Egypt) [38]. 

### 2.12. Assessment of Renal Index (Kidney Weight/Body Weight Ratio)

At the end of the experiment, the renal index was determined by measuring the animals’ body weight. The renal index was calculated by the following equation (The total weights of the left and right kidneys/the total body weight of the rats). The kidneys were removed, decapsulated from their fatty and fascial capsules, and weighed [38,39].

### 2.13. Biochemical Assay (Urine Collection and Analysis of Serum Creatinine and BUN)

At the end of the study, the animals were euthanized, and trunk blood was obtained in ice-chilled heparinized tubes. Analysis of the samples of serum was conducted for creatinine and blood urea nitrogen (BUN) using an auto Analyzer (RA 1000; Technicon Instruments, Texas, TX, USA). For urine collection, the rats were located discretely in metabolic cages for 24 h urine collection with free access to food and water. Protease inhibitors (Roche Diagnostics, Indianapolis, IN, USA) were added to prevent protein degradation. Twenty-four-hour urine samples were centrifuged to remove debris. Urinary gamma-glutamyl transpeptidase (GGT) was measured in the 24 h urine collection a day before the end of the study as a marker of tubular damage [39].

### 2.14. Assessment of the Oxidative Stress Markers in the Renal Tissue’s Homogenates

According to Beutler et al., the concentration of glutathione (GSH) was determined [40]. Superoxide dismutase enzyme (SOD) activity was estimated according to the Masayasu and Hiroshi technique [41]. The activity of catalase (CAT) was measured according to Aebi method [42]. In addition, the levels of malondialdehyde (MDA) were estimated according to Kei technique [43]. A commercial ELISA kit (MyBioSource Co., San Diego, CA, USA) was used to measure total antioxidant capacity (TAC). The procedures set forth by the manufacturer were followed at every step.

### 2.15. Biochemical Assay of the Pro-Inflammatory Markers

To estimate the inflammatory markers in the homogenate of renal tissue, ELISA commercial kits (MyBioSource Co., San Diego, CA, USA) of rats were used. Interleukins 6 and 1β (IL-6 and IL-1β) and tumor necrosis factor-α (TNF-α) were estimated. 

### 2.16. The Analysis of the Expression of Renal Genes 

The harvest of the total renal RNA was conducted by utilizing Sepasol reagent (Nacalai Tesque, Inc., Kyoto, Japan). Then, by utilizing ReverTra Ace (Toyobo Co., Ltd., Osaka, Japan) and Random Primer (Thermo Fisher Scientific, Inc., Waltham, MA, USA), cDNA first-strand was carried out. To perform the steps of the quantitative real-time polymerase chain reaction (qRT-PCR), Step One Plus Real-Time PCR with Fast SYBR Green Master Mix Reagent (Thermo Fisher Scientific, Inc., Waltham, MA, USA) were utilized. Caspase-3, Keap1, NF-κB, Nrf2, PPARγ, HO-1, and β-actin primers (Table 1) were selected to be assessed, purchased, and custom-made by (Thermo Fisher Scientific, Inc., Waltham, MA, USA). According to the Livak and Schmittgen method, the comparison CT technique was utilized for determination of the relative gene expression [44]. As an endogenous reference gene, β-actin was utilized. 

### 2.17. Statistical Analyses 

The results of the current experiment were collected and analyzed using Statistical Package for Social Sciences (SPSS version 23.0), and the data were reported as Mean ± standard deviation (SD). A one-way analysis of variance (ANOVA) was used in quantitative data. Statistical significance between groups was calculated by Tukey’s post hoc test. Statistically significant differences are represented as *p* < 0.05. Charts were drawn up using the GraphPad Prism 7.00 software. Boxplot graphs illustrated the mean values of the measured renal parameters, histopathological, immunohistochemical scores, and renal index. 

## 3. Results

### 3.1. Results of Biochemical Assay and Renal Function

**Figure 1:**
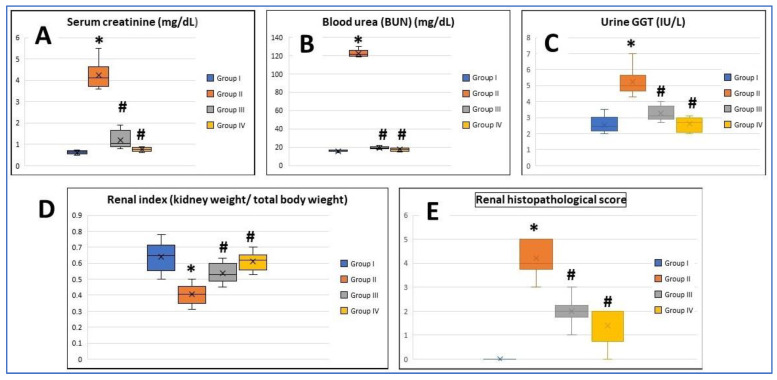
Boxplot graphs showing mean values of (**A**) serum creatinine, (**B**) blood urea nitrogen (BUN), (**C**) urine gamma-glutamyl transpeptidase (GGT), (**D**) renal index, and (**E**) renal histopathological score in all the study groups. Group I: (Control), Group II (MTX: Methotrexate), Group III: (MTX + PRP: Methotrexate +Platelet rich plasma), Group IV (MTX + MSC: Methotrexate + Mesenchymal Stem cells). *: Significant compared to group I, #: significant compared to group II, *p* < 0.05, n = 8 rats/group.

Treatments of rats with low-dose MTX once a week for 8 consecutive weeks caused a significant increase in both serum creatinine and blood urea concentration compared to the control values. PRP injection and AD-MSCs transplantation of the group III and IV rats, respectively, resulted in a significant decrease in serum creatinine and urea compared to the group II rats (*p* < 0.05) (Figure 1A,B); moreover, a significant increase in urinary GGT was observed in the group II rats compared with the normal rats. PRP managed to decrease concentration of GGT in urine, but it was still higher than those of the control group. Rats treated with AD-MSCs showed almost the same GGT concentration as the control values (Figure 1C).

### 3.2. Assessment of Renal Index

The renal index was significantly decreased and recorded in the MTX group compared to the control group (*p* < 0.05), while restoration of the control values was noticed in both treated groups (groups III and IV), and this was statistically significant (*p* < 0.05). There was no significant difference between group III and group IV regarding this parameter (Figure 1D).

### 3.3. Morphological and Phenotypic Characterization of the Isolated AD-MSCs

**Figure 2:**
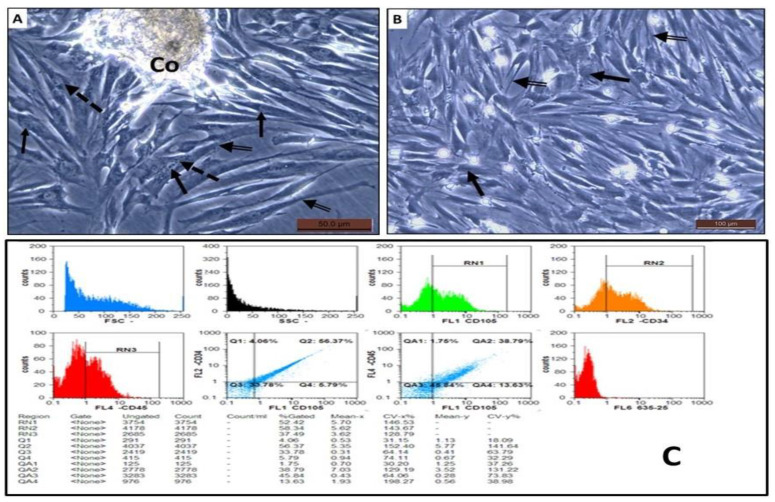
(**A**,**B**) Phase contrast of AD-MSCs isolated from rat omental fat during P0 and P3, respectively. The cells had large nuclei with multiple nucleoli (arrow), granular cytoplasm (dashed arrow) with many cytoplasmic processes (double arrow). The cells showed a tendency to form colonies (Co) with a large number of overlapping cells. (**C**) Flow cytometric analysis of AD-MSCs expression of CD 105, CD 34, and CD 45.

The isolated seeded cells from SVF of the rat omental fat were recognized to form two different population during P0: a floating cell population that was discarded with the first exchange of media and a plastic-adherent cell population that attached to the floor of the tissue culture vessels. The attached cells showed fibroblast-like morphology, that is, large nuclei with many nucleoli and granular cytoplasm were present. They also showed a high tendency to form large colonies with many overlapping cells (Figure 2A). The attached cells in the subsequent passages showed the same morphology with more homogeneity and longer cytoplasmic processes (Figure 2B). Flow cytometric analysis of the P3 cells showed that most of the cells exhibited the MSC marker CD-105, while a small number of them expressed the hematopoietic stem cell markers CD-34 and CD-45 (Figure 2C).

### 3.4. Histopathological Results

#### 3.4.1. Renal Section Stained with H&E

**Figure 3:**
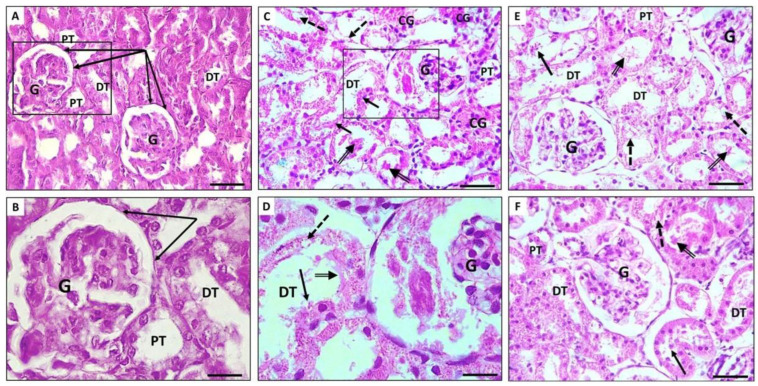
Photomicrographs showing H&E-stained sections of rat kidney. (**A**,**B**) Renal section of control group showing normal histological structure with intact glomeruli and renal tubules. (**C**,**D**) Renal sections of MTX group showing massive tubular dilatation, hyaline casts (double arrow), and tubular epithelial vacuolation (dashed arrow) and desquamation (arrow). Interstitial congestion (CG) and glomerulosclerosis were also evident. (**E**,**F**) Renal sections of PRP and AD-MSCs groups, respectively, show restoration of glomerular and tubular architecture; however, some vacuolation (dashed arrow) and desquamation of tubular epithelium (arrow) were still seen. Glomerulus (G), distal convoluted tubules (DT), proximal convoluted tubules (PT), Bowman’s capsules (collected arrows). The squared parts in (**A**,**C**) are magnified in (**B**,**D**), respectively. Scale bar in (**A**,**C**,**E**,**F**) 50 µm. Scale bar in (**B**,**D**) 20 µm.

Renal tissues revealed normal histological findings in the control group with intact glomeruli (G), Bowmans capsules (BC), and proximal and distal convoluted tubules (PT and DT, respectively) (Figure 3A,B). In the MTX group, there were significant changes mainly in the renal tubules in the form of tubular necrosis, large hyaline casts, and tubular epithelial vacuolation and desquamation. Additionally, tubular dilatation and interstitial congestion were obviously seen. On the other hand, many glomeruli exhibited glomerulosclerosis with widening of Bowman’s space (Figure 3C,D). Renal changes in the MTX group nearly regressed to normal in the treated groups with PRP and AD-MSCs (Figure 3E,F, respectively). No significant differences in the microscopic finding between the PRP- and AD-MSCs-treated groups were detected. Both groups showed almost complete restoration of the glomerular and tubular structure apart from some small vacuolation and desquamation of the tubular epithelium that were still recognized in few specimens. The renal histopathological score was significantly higher in group II in comparison to the control rats (*p* < 0.001) and was significantly reduced and approaching the control value in rats treated with PRP (group III) and AD-MSCs (group IV) (*p* < 0.05). Groups III and IV showed no significant difference (Figure 1E).

#### 3.4.2. Masson-Trichrome Stained Sections

**Figure 4:**
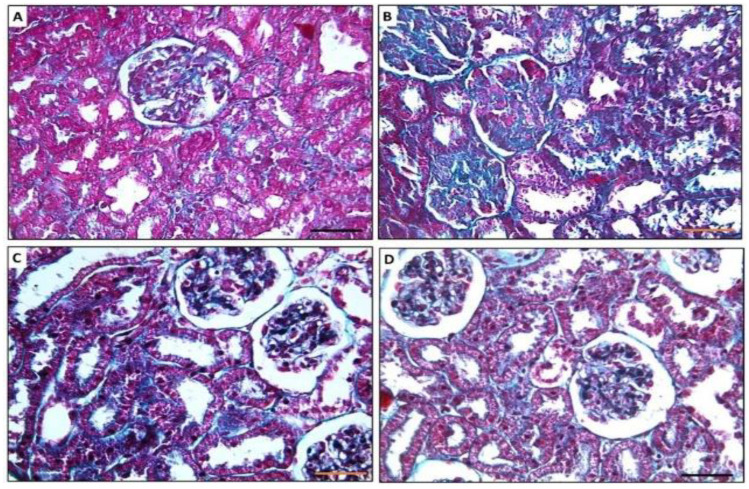
Photomicrographs showing Masson-trichrome-stained sections of rat kidney. (**A**) Renal section of control group showing minimal collagen fibers in interstitial tissue and glomeruli. (**B**) Renal sections of MTX group showing massive fibrosis both in the glomerular tuft areas and in the interstitial tissue. (**C**,**D**) Renal sections of PRP and AD-MSCs groups, respectively, showing few collagens fibers deposited in the interstitial tissue and minimal deposition in the glomeruli. ×400, scale bar 50 µm.

The renal tissue of the MTX-treated group (Figure 4B) exhibited massive glomerular and interstitial fibrosis compared to the control (Figure 4A) kidneys. The fibrosis decreased markedly in the glomeruli and in the interstitium of both groups III (Figure 4C) and IV (Figure 4D) with obvious improvement in the glomeruli. 

#### 3.4.3. Periodic Acid Schiff (PAS)-Stained Sections

**Figure 5:**
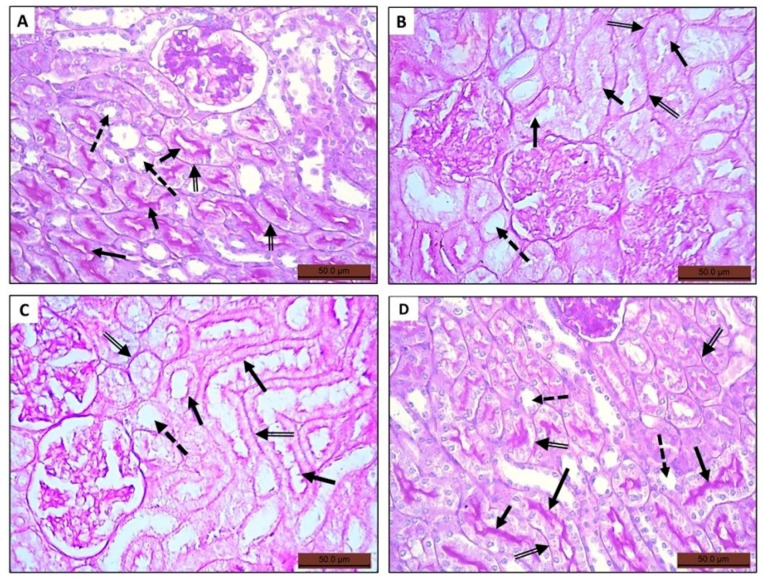
Photomicrographs of histological sections stained with periodic acid Schiff (PAS) of rat kidney of the tested groups. (**A**) Renal section of control group depicting expression of PAS-positive material in glomerular mesangial matrix, the basement membrane (double arrow), and the cilia of the epithelium lining the proximal convoluted tubules (arrow). (**B**) Renal section of MTX group showing tubular alteration, thinning, interruption of basement membranes (double arrow), and lack of PAS-positive material expression within the tubular epithelium (arrow). (**C**,**D**) Renal section of groups III and IV, respectively, showing restoration of the tubular epithelium in group III but clearly seen in group IV indicated by the PAS-positive stained brush border (arrow). All groups showing lack of PAS-positive material expression in the epithelium of the distal convoluted tubules (dashed arrow).

Examination of the renal tissue stained with PAS from the control group revealed expression of PAS-positive material in the glomerular mesangial matrix, tubular basement membrane, and brush border of proximal convoluted tubular epithelium (Figure 5A). The treated rats with MTX showed marked disintegration, gaps with thinning of the tubular basement membranes, together with destruction of the cilia of the PT epithelium (Figure 5B) in their renal tissue versus the control group. Almost normal tubular and glomerular architecture were preserved in groups III and IV with apparent better improvement in group IV (Figure 5C,D, respectively). 

#### 3.4.4. Immunohistochemically Stained Sections

##### Expression of Casp-3

**Figure 6:**
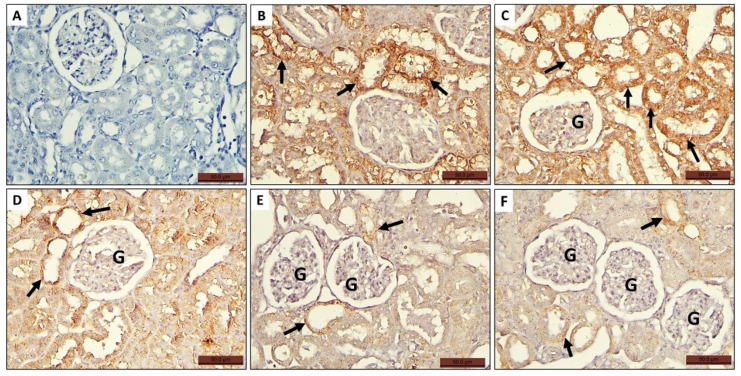
Photomicrographs of histological sections of rat kidney of all groups stained with anti Casp-3 antibodies showing expression of the antibodies in some glomerular cells (G) and in the tubular epithelium (arrow). (**A**) Control group, (**B**,**C**) MTX group, (**D**) MTX + PRP group, and (**E**,**F**) MTX + MSC group.

The expression of Casp-3 antibodies in the renal tissue was observed in very few, if any, glomeruli and tubular epithelium of the control group (Figure 6A). Its expression markedly increased in the MTX group (Figure 6B,C) compared to the control group and regressed near the control values in both groups III (Figure 6D) and IV (Figure 6E,F) with even more apparent regression in their expression in group IV compared to group III.

##### Expression of iNOS 

**Figure 7:**
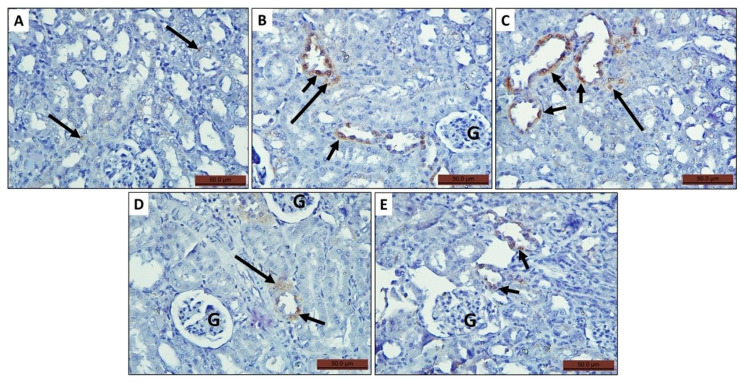
Photomicrographs of histological sections of rat kidney of the tested groups stained with immunohistochemical stain of iNOS antibodies showing expression of the antibody in some tubular epithelium (short arrow) and interstitial spaces (long arrow). (**A**) Control group, (**B**,**C**) MTX group, (**D**) MTX + PRP group, and (**E**) MTX + MSC group.

The Expression of iNOS antibodies in the renal tissue was observed in very few specimens of the control group, and in few tubular epithelium, perivascular, and interstitial spaces (Figure 7A). Its expression markedly increased in the MTX group (Figure 7B,C) compared to the control group and lapsed near the control values in both groups III (Figure 7D) and IV (Figure 7E) with apparent more lapsing in their expression in group IV compared to group III. 

### 3.5. Morphometric Image Analysis Results

#### 3.5.1. Glomerular Mesangial Index

**Figure 8:**
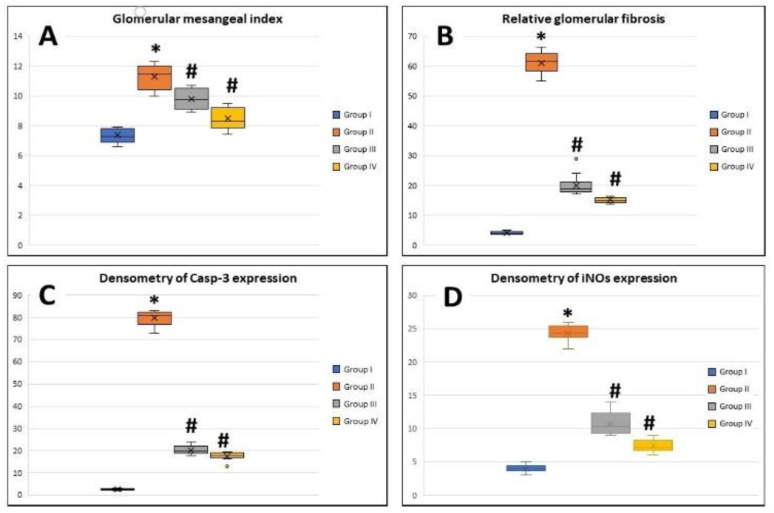
Boxplot graphs showing mean values of glomerular mesangial index. (**A**) Relative glomerular fibrosis, (**B**) densitometry of Casp-3 expression, (**C**) and densitometry of iNOS expression (**D**) in renal tissues of all the study groups. Group I: (Control), Group II: (MTX: Methotrexate), Group III: (MTX + PRP: Methotrexate +Platelet rich plasma), Group IV (MTX + MSC: Methotrexate + Mesenchymal Stem cells). *: Significant compared to group I, #: significant compared to group II, *p* < 0.05, n = 8 rats/group.

The mesangial index exhibited a significant increase in the MTX group (11.2 ± 0.7%) compared to the control group (7.3 ± 0.4%) (*p* < 0.05). PRP- and AD-MSCs-treated groups showed a significant decrease in mesangial index (9.76 ± 0.6% and 8.4 ± 0.7%, respectively) compared to MTX-treated rats (*p* < 0.05) with no significant difference between them (Figure 8A).

#### 3.5.2. Relative Glomerular Fibrosis

The deposition of massive collagen fibers and renal fibrosis were detected in the MTX-group sections stained with Masson trichrome. The relative glomerular fibrosis in control group was 4.02 ± 0.4% and 61.7 ± 3.4% in the MTX group with highly significant difference between them (*p* < 0.001). The PRP- and AD-MSCs-treated groups showed a significant decrease in relative glomerular fibrosis (20.09 ± 3.5% and 15.1 ± 0.8%, respectively) compared to MTX-treated rats (*p* < 0.05) with no significant difference between them (Figure 8B).

#### 3.5.3. Densitometry of Casp-3 Expression

The densitometric expression of Casp-3 antibodies in renal tissue revealed a significant increase in its optical density in the MTX group (79.7 ± 3.08) compared to the control group (2.6 ± 0.3) (*p* < 0.001), while its expression in groups III and IV decreased significantly (20.2 ± 1.8 and 17.5 ± 1.8, respectively) compared to the MTX group (*p* < 0.005). There was no significant difference between groups III and IV (Figure 8C).

#### 3.5.4. Densitometry of iNOS Expression

The densitometric expression of iNOS revealed an increase in the optical density of iNOS significantly in MTX-treated rats (24.1 ± 1.1) compared to the control group (4.05 ± 0.5) (*p* < 0.05), while its expression in the PRP- and AD-MSCs-treated groups (10.7 ± 1.7 and 7.4 ± 0.9, respectively) decreased significantly (*p* < 0.05) compared to MTX-treated rats. Groups III and IV showed no significant difference (Figure 8D).

### 3.6. Ultrastructure Assessment of Renal Tissue

**Figure 9:**
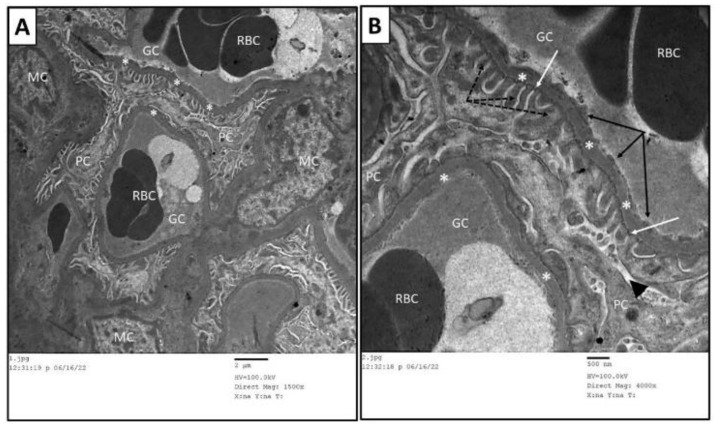
Transmission electron micrographs of glomerular capillary loops of control group. (**B**) is a higher magnification of (**A**). The glomerular capillaries (GC) had intact basement membrane (*) and were lined by fenestrated endothelial cells (black arrows). The urine side of the capillaries was embraced by foot processes (dashed arrow) of podocytes (PC). Slit diaphragm (white arrow) was located between each adjacent foot processes. The mesangium is located centrally among capillary loops and contains mesangial cells (MC). RBC: red blood cells.

**Figure 10:**
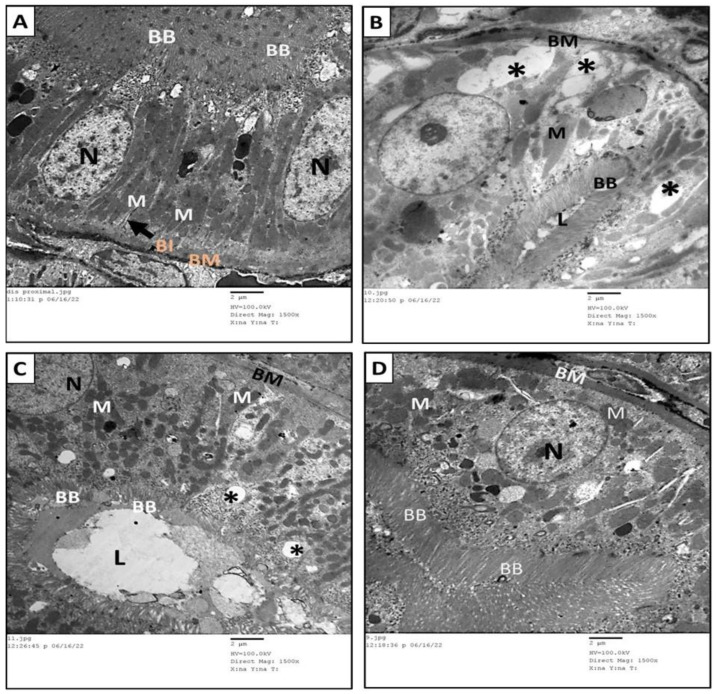
Transmission electron micrographs of proximal convoluted tubules of the different groups in the study. (**A**) The PT of the rats in the control group showed normal structure. (**B**) The PT of MTX group showed massive vacuolations (*), short, interrupted brush border (BB), loss of basal infoldings of the basement membrane, and decreased number and disorganization of mitochondria (M). (**C**) The PT of MTX + PRP group showed moderate restoration of normal structure apart from few vacuolations (*) and poorly formed brush border (BB). (**D**) The PT of MTX + MSC group showed great restoration of normal structure compared to control group. BM: Basement membrane, BI: basal infoldings, M: mitochondria, L: lumen, N: nucleus, BB: brush border and *: vacuolations.

Transmission electron microscopy of the renal cortices of the control group showed normal glomerular and tubular structure. The renal cortices had multiple glomeruli, each consisted of tuft capillaries. The wall of each glomerular capillary was formed by the glomerular basement membrane (GBM) lined with fenestrated endothelial layer. The GBM was surrounded by a discontinuous layer of podocytes; each had a cell body, from which extended numerous foot processes incorporating the glomerular capillaries. The processes of podocytes were interdigitated. Elongated spaces, called filtration slits, were found between the neighboring foot processes. Mesangial cells were evident between capillaries (Figure 9A,B). The PT were lined with cuboidal cells and exhibited microvilli or brush border extending from the apical portion towards the lumen. The cytoplasm of these cells contained numerous spherical or elongated mitochondria. The tubular cells rested on a tubular basement membrane, which in turn showed multiple infoldings to increase the cell surface at the basal regions (Figure 10A). 

**Figure 11:**
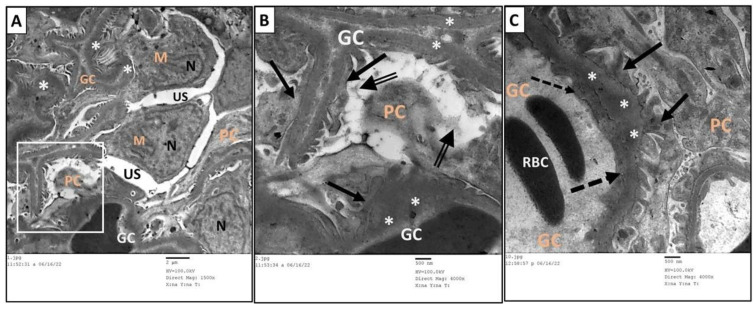
Transmission electron micrographs of glomerular capillary loops of MTX group. (**B**) is a higher magnification of the squared part in (**A**). The glomerular capillaries (GC) were surrounded by wide urinary space (US). Podocytes showed fused foot processes (arrow) and faint rudimentary ones (double arrow). Irregular nuclei (N) of mesangial cells (M) were noticed. Thickened basement membrane (*) was clearly seen in panel **(C)**. RBC: red blood cells.

**Figure 12:**
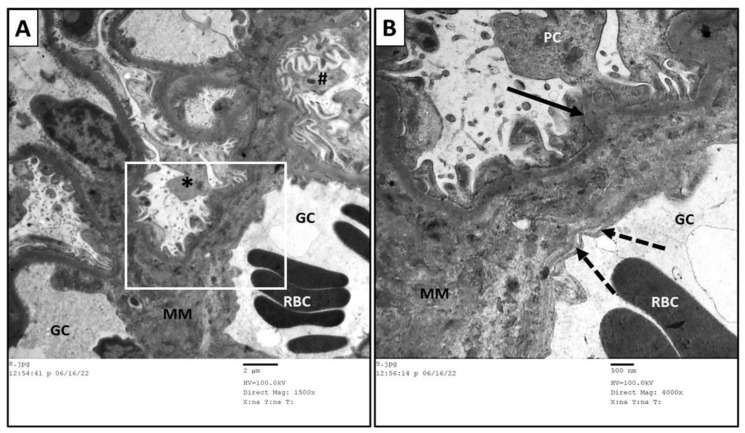
Transmission electron micrographs of glomerular capillary loops of MTX + PRP group. (**B**) is a higher magnification of the squared part in (**A**). The glomerular capillaries (GC) showed well-formed and arranged podocytes in many segments (#). Focal affection was seen in other segments (*) in which the podocytes (PC) exhibited fused foot processes (arrow) and splitting of the basement membrane (dashed arrow). An amorphous mesangial matrix (MM) was located between the capillary loops. RBC: red blood cells.

**Figure 13:**
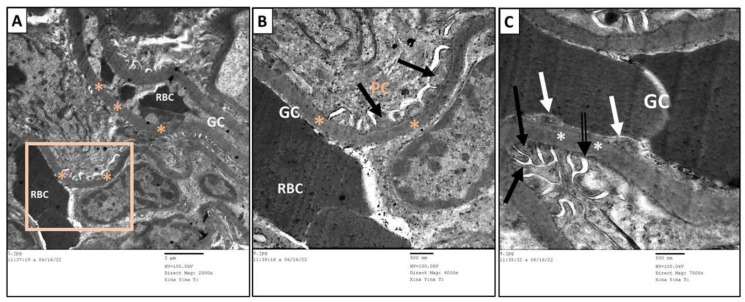
Transmission electron micrographs of glomerular capillary loops of MTX + MSC group. (**B**) is a higher magnification of the squared part in (**A**). There was normal structure of the glomerular capillaries (GC), basement membrane (*), foot processes (black arrow) of podocytes (PC), slit diaphragm (double arrow), and fenestrated endothelial layer (white arrow) was obviously appeared in (**C**).

The renal cortices of the MTX group revealed thickening of basement membrane of many glomerular capillary loops and fusion (effacement) of podocyte foot processes. In some capillary tufts, the podocytes exhibited very faint foot processes that appeared as fine rudimentary cytoplasmic extensions. The endothelial cell lining of the glomerular capillary appeared edematous and lacked the fenestrated pattern of their arrangement. Widening of the urinary space within the Bowman’s capsule was also evident (Figure 11A,B). The PT of this group exhibited numerous cytoplasmic vacuolations, cytoplasmic organelles’ disorganization, brush border shortness, irregularity, and interruption. Decreased number of mitochondria and loss of their basal arrangement were clearly displayed along with inhomogeneity of the basement membrane and loss of the basal infoldings (Figure 10B). The ultrastructural changes in the MTX group improved significantly in groups III and IV with better improvement in group IV. Normal structure of glomerular capillary basement membrane, sound arrangement of the podocytes’ foot processes, and intact capillary endothelium with a normal fenestrated arrangement pattern were seen in almost all of the specimens of groups III (Figure 12) and IV (Figure 13), while a few specimens in group III were still showing focal splitting of the basement membrane of the glomerular capillary along with fusion of a few podocytes foot processes (Figure 12). 

These ultrastructure of the PT of both groups III and IV (Figure 10C,D) revealed the restoration of their normal structure in comparison with the control group. Furthermore, some vacuolation and disorganized short brush border of the tubular epithelium persisted in a few specimens of group III (Figure 10C).

### 3.7. AD-MSCs and PRP Alleviate Kidney Oxidative Stress in MTX-Induced Renal Damage

To assess how MSC and PRP might affect and alleviate the damage produced from the oxidative stress in renal tissue, we estimate the marker levels of lipid peroxide (MDA), antioxidant enzyme activity (SOD, CAT, GSH), and TAC (Table 2). The activities of SOD, CAT, GSH and TAC showed significant decreases in the rats of the MTX group which was accompanied by a significant increase in the MDA levels compared with the levels in rats of the control group (*p* < 0.05). MSC or PRP administration promoted the activities of the antioxidant enzymes and declined the levels of MDA compared to the MTX-treated group (*p* < 0.05). The current finding proves that MSC and PRP recovered the antioxidant capacity in renal tissue of rats. Furthermore, no significant differences were detected in their activities comparing the two co-treatment groups.

### 3.8. AD-MSCs and PRP Mitigate Renal Inflammatory Response in MTX-Induced Renal Injury

The renal pro-inflammatory cytokines IL-1β, IL-6 and TNF-α levels were significantly elevated in rats injected with MTX (*p* < 0.05) compared to normal rats in control group. However, MSC and PRP co-treatment with MTX showed significant decrease in the renal inflammatory markers levels versus the MTX-treated rats (*p* < 0.05, Table 3).

### 3.9. MTX and PRP Adjust the Expression Levels of Renal Gene in MTX-Intoxicated Rats 

**Figure 14:**
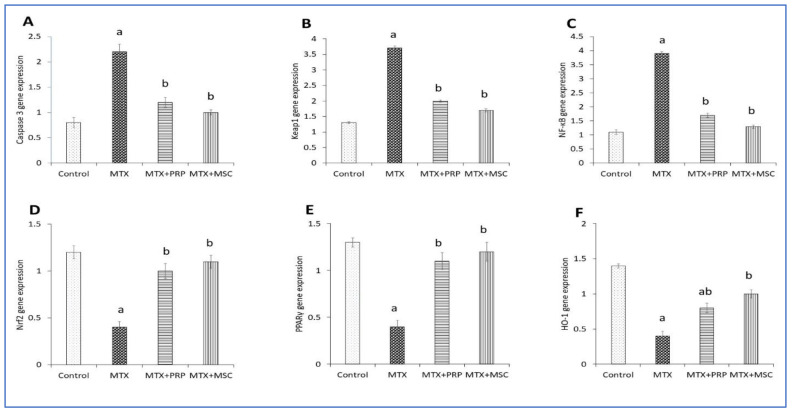
Effects of MSC and PRP on the expression levels of renal gene in MTX-intoxicated rats. Representative graphs of genes expression level analysis show caspase-3 mRNA. (**A**) Keap1 mRNA, (**B**) NF-κB mRNA, (**C**) Nrf2 mRNA, (**D**) PPARγ mRNA, (**E**) HO-1 mRNA, and (**F**). MTX: methotrexate; PRP: platelet rich plasma; HO-1: heme oxygenase-1; Keap1; Kelch-like ECH-associated protein 1; NF-κB: nuclear factor kappa-light-chain-enhancer of activated B cells; Nrf2; nuclear factor erythroid 2-related factor 2; and PPARγ: Peroxisome proliferator-activated receptor gamma. Data are expressed as mean ± SD. a, b: Significantly different from normal control and MTX groups, respectively, at *p* < 0.05.

To figure out whether MSCs and PRP could affect the Nrf2/PPARγ/HO-1 and NF-κB/Keap1/caspase-3 signaling pathways in rats with MTX toxicity, mRNA expression levels of Nrf2, HO-1, Keap1, PPARγ, NF-κB, and caspase-3 were assessed. The expression analysis of the selected gene in rats treated with MTX depicted a significant upregulation (*p* < 0.05) of caspase-3, Keap1, and NF-κB mRNA expression levels compared to the rats in the control group; however, rats treated with MSCs and PRP showed significant downregulation of these molecular changes compared to the rats in the MTX group (Figure 14A–C). The renal expression levels of genes (HO-1, Nrf2, and PPARγ) showed significant decline in rats treated with MTX compared to rats in the control group. Furthermore, MSC and PRP co-treatment upregulated the expression levels of Nrf2, PPARγ, and HO-1 genes compared to rats treated with MTX (Figure 14D–F). The current findings proposed that the signaling pathways of Nrf2/PPARγ/HO-1 and of NF-κB/Keap1/caspase-3 could be prompted in rats treated with MSCs and PRP. These findings proposed that MSCs and PRP might prevent apoptosis caused by oxidative stress.

## 4. Discussion

MTX is a wonderful choice for various clinical utilization, as it has a wide safety data, low cost, and regular treatment regimens [45]; however, these characteristics hide behind the harmful and toxic impact on many organs in the body, especially the kidneys [13]. The mechanism of MTX induced nephrotoxicity and involves the induction of oxidative stress and inflammation [46,47]. In the current study, we investigated the effects of AD-MSCs and PRP supplementation on MTX-induced nephrotoxicity in rats. The potential mechanisms might involve suppressing inflammatory responses, alleviating oxidative stress, activating the Nrf-2/HO-1 pathway, and improving the destruction of renal tissue. 

In the present study, a considerable increase in the levels of serum urea and creatine was demonstrated in the MTX-administered group which alleviated with MSC and PRP co-treatment. Urea and creatine are considered beneficial quantitative parameters for assessing renal injury [47].

The disequilibrium between oxidative and antioxidant parameters has a crucial role in separating numerous harmful triggers alongside its significance for estimating the degree of tissue injury [48]. In the current study, the authors observed significantly higher tissue MDA concentration in MTX-injected group, whereas co-treatment with either MSCs or PRP + MTX significantly decreased the concentration of MDA in renal tissue. In this context, the formation of oxidative aggressions can damage macromolecules and ultimately cause harmful oxidative effects on the cellular components [49]. In addition, MTX enhances disruption of the defense system of mitochondria, shackling its function and therefore increases the levels of cellular oxidative damage. These recent studies support the notion that MTX administration might prompt renal insults through oxidative stress induction [47]. The levels of antioxidants such as SOD, GSH, CAT, and TAC in renal tissue of the MTX-treated rats were significantly decreased, whereas the co-administration of either MSCs or PRP + MTX increased these antioxidant enzymes. Similar changes were observed in the oxidant and antioxidant profiles by Gad et al. in their study [27]. In a meta-analysis done by Lin S et al. who assessed the nephroprotective effects of MSCs in kidney diseases induced by toxicants, they observed that the oxidative stress markers were decreased, and antioxidants were increased upon administration of MSCs [50]. This may be elucidated by the declined level of NOX4 expression and Nrf2 transportation from cytoplasm to the nucleus with stimulation of ARE signaling of Nrf2, which suppresses the oxidative stress generation.

The present study centered around the critical role of Nrf2 in conditions of oxidative stress and, in our case, MTX toxicity. Nrf2 belongs to the cap ‘n’ collar-basic leucine zipper family, which is expressed in the kidney, and it is a redox-dependent transcription factor which promotes antioxidant enzyme production in times of oxidative stress and inhibits the process of apoptosis [51,52]. Nrf2, which is normally coupled to Keap1 and is confined to the cytoplasm, separates from it in times of oxidative stress and relocates to the nucleus. In the nucleus, Nrf2 binds to the antioxidant response elements (ARE) and affects the gene expression of antioxidant and detoxifying enzymes [53,54,55]. One of the important enzymes is the phase II detoxification enzymes such as HO-1 (downstream enzyme), SOD, CAT, and GPx, which play an important role in protecting the kidney against oxidative stress and inflammation. MTX-induced renal damage has been attributed to the increased levels of MDA and caspase-3 expression and decreased levels of HO-1 and Nrf2 expression [13]. The present study revealed that MTX-treated rats had significantly decreased glutathione levels, Nrf2 and HO-1 expression, as well as increased MDA levels; however, the changes were reversed upon pretreatment with MSCs and PRP which resulted in decreased MDA and increased Nrf2, HO-1, and glutathione levels, signifying their potential therapeutic effect. The signaling pathway of Nrf2/HO-1 is deemed to be the major safeguard mechanism against the injury and damage of oxidative stress [56,57].

HO-1, an inducible stress response protein, is a part of an integral antioxidant system, which is regulated by Nrf2. It helps in catalyzing the oxidative process of heme breakdown to form biliverdin and exerts cytoprotective effects in oxidative stress [58]. Our results depicted that treatment with MSCs and PRP results in dissociation of Nrf2 from the Nrf2–Keap1 complex which facilitates its entry into the nucleus thereby boosting the expression of antioxidant enzymes. There appears to be a tight multilayered regulation of the signaling system of Keap1–Nrf2–ARE [59]; therefore, weakening of the oxidant mechanisms via activation of the Nrf2/HO-1 pathway appears to be one of the important mechanisms through which MSCs and PRP are able to reverse the toxicity induced by MTX.

Alongside the renal oxidative stress, there is evidence that inflammation plays a crucial role in the mechanisms that induce nephrotoxicity. In order to determine the inflammatory response in rats treated with MTX, the current study investigated the sensitive and accurate cytokines expression levels of tissue pro-inflammatory markers IL-1β, IL-6, and TNF-α. Renal pro-inflammatory markers such as IL-1β, IL-6, and TNF-α levels were elevated in MTX-injected group; however, MSC and PRP co-treatment with MTX depicted significant reduction in the levels of these inflammatory markers. These findings indicate the anti-inflammatory effects exerted by MSCs that promote the recovery of injured renal cells and may restore renal function. This ameliorative effect may be related to decline in the expression of NF-κB levels in addition to the suppression of peroxynitrite synthesis. These alterations cause NF-κB deactivation, which leads to decline in the expression level of the pro-inflammatory mediators [60]. In a meta-analysis done by Lin S et al., they observed that MSC administration decreased the expression of pro-inflammatory cytokines such as TNF-α, IFN-γ, and IL-1β as well as increased the expression of anti-inflammatory cytokines such as IL-1, IL-10, etc. [50]. As PRP is a rich source of growth factors, interleukins, and other cytokines, this explains the anti-inflammatory effect in the PRP-treated group [61,62]. Upon analysis of 10 independent human samples, a total of 1507 unique proteins in platelets were identified by Qureshi et al. [63]. PRP is considered a contributor to tissue healing and regeneration, as it is a pool of biologically active autologous components [64]; furthermore, numerous growth factors were detected within PRP, including VEGF [65]. Such factors activate the cellular signaling pathway, the crucial cell survival pathway in renal cells.

MTX treatment induced inflammatory response in the renal tissue which was evidenced by increased levels of inflammatory markers such as NF-κB, IL-1β, and TNF-α in the renal tissue in comparison to the control group; however, the inflammatory markers were considerably reduced in the MSC- and PRP-treated groups in comparison to the MTX-treated group. The anti-inflammatory effects exerted by MSCs and PRP are partially mediated by activation of PPARγ, which are a part of nuclear hormone receptor superfamily, whose activation controls gene expression in metabolic and inflammatory pathways [66]. PPAR are transcription factors and there are three subtypes: PPARα, PPARγ, and PPARβ/δ, out of which PPARγ is expressed in high concentrations in the kidney tissue where it controls the level of inflammatory activity [67]. We observed significantly decreased PPARγ mRNA levels in MTX-treated renal tissue, whereas treatment with MSCs and PRP significantly increased the PPARγ expression, thereby exerting anti-inflammatory effects.

Platelet-rich plasma (PRP) is derived from plasma by fractionation and contains more platelets than whole blood [68]. PRP is rich in many cytokines, and important growth factors such as TGF-ꞵ1, PDGF, insulin-like growth factors, PF-4, FGF-2, and VEGF [69,70]. These growth factors and cytokines contribute to the possible therapeutic effect of PRP by recruiting the resident stem cells to the site of injury followed by their activation and production of more cytokines and growth factors [71]. Furthermore, growth factors in PRP increase stem cell proliferation and differentiation, promote angiogenesis, as well as lead to increased formation of extracellular matrices [72]. Platelets in PRP exert inhibitory effects on the NF-κB pathway, thereby decreasing the inflammation [73].

The expression of the renal gene was assessed using qRT-PCR analysis and revealed significant increase in caspase-3, Keap1, and NF-κB mRNA expression levels in the MTX-treated rats, and these changes were significantly decreased by MSC and PRP co-treatment. Similar results were obtained by Gad AM et al. in their study where they observed increased levels of caspase-3 and NF-κB in MTX-treated rats, whereas BM-MSC and AD-MSC administration decreased these levels [27]; however, HO-1, Nrf2, and PPARγ gene expression was significantly declined in the rats treated with MTX, whereas MSC and PRP co-treatment caused remarkable rising in HO-1, Nrf2, and PPARγ genes expression levels. In a study done by Abd El-Twab SM et al. on the effects of chicoric acid on the prevention of methotrexate-induced kidney injury, they observed MTX-induced rats exhibited decreased expression of Nrf2, NQO-1, and HO-1. Chicoric acid administration suppressed the expression of NF-κB p65, NLRP3, caspase-1, and IL-1β while inhibiting apoptosis as revealed by increased Bcl-2 gene expression and decreased expression of Bax and caspase-3 [74]. Mahmoud AM et al. observed the beneficial effects of ferulic acid on MTX-induced toxicity in rats, wherein MTX caused apoptosis leading to increased expression of Bax, cytochrome c, and caspase-3, and decreased Bcl-2, whereas ferulic acid reversed these effects. In addition, FA-treated groups showed increased expression of Nrf2/ARE/HO-1 signaling and PPARγ expression [75]. All the above-mentioned findings indicate that MTX promotes inflammation and apoptosis as evidenced by the increased levels of pro-inflammatory genes and pro-apoptotic markers.

Our finding depicted that the expression of caspase-3 in rats treated with MTX was increased in renal tissue, which explains its role in apoptosis induction. Caspase-3 has a major role in the apoptotic cascade as a promoter and terminal effector; however, this caspase-3 expression was declined in MSC and PRP administration with MTX as well as reduction in the percentage of caspase-3 positive cells. The signaling pathway modulation is considered one of the main mechanisms that explains apoptosis reduction, which also boosts cell development and promotes cell proliferation. The current results depicted the role and effect of MSCs and PRP as antiapoptotic agents which is evidenced by alleviation of caspase-3 expression. Cell apoptosis could be initiated by ROS by the upregulation of Bax and caspase-3 which serves as pro-apoptotic proteins. Diverse enzymes from antioxidant systems can inhibit the previous proapoptotic proteins [76,77].

Caspase-3 constitutes one of the basic enzymes required for both the extrinsic and intrinsic pathways of apoptosis [78]. Our study revealed that MTX treatment caused considerable surge in expression of caspase-3 gene levels, thereby resulting in pronounced apoptosis in the renal tissue. This may be attributed to changes in the permeability of the mitochondrial membrane; the release of cytochrome c into the cytosol, thereby promoting apoptosome formation; and the leading to the activation of caspase-3 [79]. Co-treatment with PRP and MSCs depicted significantly decreased levels of caspase-3 expression, and this is in line with the findings of earlier studies [80,81]. Our findings delineated the capability of the PRP and MSCs to abate apoptosis within the injured renal tissue, thereby confirming their antioxidant and anti-inflammatory properties.

**Scheme 2:**
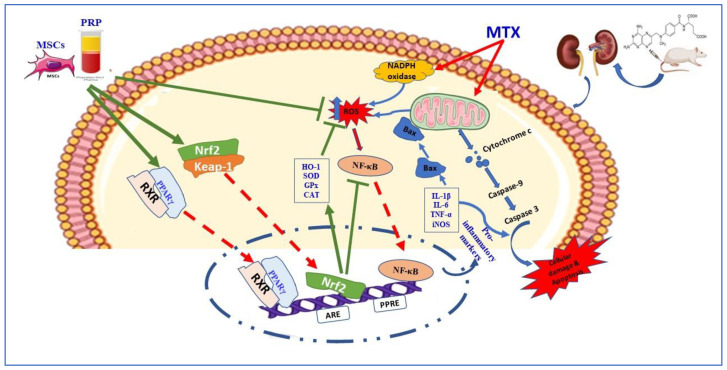
A proposed schematic diagram illustrating the protective mechanism of AD-MSCs against MTX-induced renal toxicity. MTX: methotrexate; ROS: reactive oxygen species; Nrf2: nuclear factor (erythroid-derived 2)-like 2; NF-κB: nuclear factor-kappa B; Bax: Bcl-2-associated X protein; Keap-1: Kelch-like ECH-associated protein 1; ARE: antioxidant response element; HO-1: Heme oxygenase-1; PPARγ: peroxisome proliferator activated receptor gamma; RXR: retinoid X receptor.

The present study results elucidated the crucial pathways involving Nrf2, NF-κB, HO-1, and PPARγ [82]. NF-κB regulates the formation of inflammatory cytokines [13]. PPAR γ upregulates the antioxidant enzymes [83] and suppresses the NF-κB and NADPH oxidases [84]. HO-1 also exerts prominent antioxidant effects [85]. Our results are in line with previous studies and depicted that MTX significantly decreased PPARγ, HO-1, and Nrf2 gene expressions, whereas it caused significant elevations in Keap1 and NF-κB gene expression [86]. MSC and PRP administration significantly regressed these molecular changes in the kidney tissue, thereby affirming their antioxidant and anti-inflammatory roles [87] (Scheme 2).

In the current study, we observed the morphological and phenotypic characteristics of the isolated seeded cells from SVF of rat omental fat with time and passages and that the cells showed the same morphology but with more homogeneity and longer cytoplasmic processes; the flow cytometric analysis exhibited the mesenchymal stem cell marker CD-105, and only a few cells expressed the hematopoietic stem cell markers CD-34 and CD-45. Our observations met the criteria as defined by the International Society for Cell Therapy (ISCT) [88], and researchers such as Czapla J et al. also observed the same morphological and phenotypic characteristics [89].

Methotrexate induces significant changes in the renal tubules such as tubular dilatation and necrosis, large hyaline casts, tubular epithelial vacuolation, and desquamation. In addition, it causes glomerulosclerosis with widening of Bowman’s space in many glomeruli. Such methotrexate-induced toxic effects in renal tubules and glomeruli have been observed in previous research [90,91,92,93]. MTX causes renal injury by increasing the formation of reactive oxygen species, which results in oxidative stress. This has been attributed to decreased formation of NADPH [94]. The other proposed mechanism is that MTX as well as its metabolite may deposit in the renal tubules, directly damaging them and leading to their necrosis [95]. 

The renal changes in the PRP and AD-MSC groups nearly regressed to normal when compared to the MTX group; however, no significant differences were observed in the histopathological findings between PRP- and AD-MSC-treated groups. Both the treated groups showed almost restoration of the glomerular and tubular structure with only small vacuolations and desquamation of the tubular epithelium at a few places. The Renal histopathological score was significantly higher in group II when compared to that of the control group but was significantly decreased and almost approaching the control value in PRP and AD-MSCs groups; however, no significant differences were observed between the two treated groups. Gad AM et al. observed in their study that MSC administration ameliorated the toxic effects induced by MTX on the renal tissue [27]. This was related to the property of MSCs to induce tissue regeneration through their immunomodulatory effects by providing soluble factors [96]. There is also evidence regarding effects being mediated via paracrine and endocrine mechanisms in addition to the direct cell–cell interactions [97]. The beneficial effects of PRP on acute renal injury have been well demonstrated by other researchers [21,98]. The protective effects of PRP are attributed to the secretion of cytokines such as interleukins, GM-CSF, and G-CSF as well as growth factors such as VEGF, basic FGF, and IGF-I [99,100]. Furthermore, the Masson trichrome stain used in our study confirmed the massive glomerular and interstitial fibrosis induced by MTX and reversal of these changes in the treated groups. Periodic acid Schiff (PAS) staining depicted the deposition of PAS-positive material in the mesangium, tubular basement membrane, and brush border of the proximal convoluted tubular epithelium along with marked disintegration and thinning of the tubular basement membranes; however, these changes were ameliorated in the treated groups.

The immunohistochemical expression of Casp-3 and iNOS antibodies was evaluated in our study. Their expression was markedly increased in the MTX group as compared to the control but regressed to near the control values in treated groups. Enhanced Casp-3 expression was observed by Gad et al. in their study in the MTX group, and decreased expressivity in the AD-MSC group was also observed. They also observed increased levels of NOx in the MTX group, whereas AD-MSC group had decreased levels [27]. Increased expression of Casp-3 indicated increased apoptotic activity in the MTX group due to increased oxidative stress [101], whereas decreased expression in the treated groups indicated decreased apoptosis. Increased NOx levels in the MTX group pointed toward increased inflammatory activity in the MTX group [102], whereas decreased levels in the treated groups signified anti-inflammatory effects of PRP and AD-MSCs.

Transmission electron microscopy of renal tissue in the MTX group depicted apoptosis of endothelial cells in the glomeruli along with vacuolation, disorganization of cytoplasmic organelles, ruptured mitochondria, and destruction of cilia of the tubular epithelium. These changes, however, improved significantly in the treated groups. Similar changes have been observed by Elsawy et al. in their study in the glomeruli and tubules in the MTX group; however, they observed that these alterations were ameliorated in their naringin-treated animals [92]. Abbas et al. in their study observed degeneration of podocytes, pyknosis of nuclei and damaged mitochondria, and cytoplasmic degeneration in the MTX group; however, the group treated with zafirlukast + MTX depicted that most of the renal tissue retained its normal morphology [103].

In the same context, morphometric image analysis for glomerular mesangial index, relative glomerular fibrosis, and densitometry of Casp-3 and iNOs expression were performed. Glomerular mesangial index exhibited a significant increase in the MTX group, whereas the PRP- and AD-MSC-treated groups depicted a significant decrease in mesangial index. For relative glomerular fibrosis analysis, massive collagen deposition and renal fibrosis were detected in the MTX group, whereas PRP- and AD-MSC-treated groups showed a significant decrease in relative glomerular fibrosis. The density of Casp-3 expression depicted a significant increase in optical density in the MTX group while its expression in groups III and IV decreased significantly. Densitometry of iNOS expression reveals a significant increase in the optical density in group II, whereas its expression in PRP- and AD-MSC-treated groups decreased significantly. All these findings from the morphometric analysis revealed increased mesangial matrix and massive fibrosis along with increased expression of Casp-3 and iNOs in the MTX group with improvement of fibrosis and decreased expression of Casp-3 and iNOs in PRP- and AD-MSC-treated groups.

## 5. Conclusions

Our study provides unprecedented information about the postulated ameliorative actions of PRP and MSCs and their ability to induce faster recovery of renal damage triggered by MTX. Low-dose MTX (1 mg/kg once weekly) administration resulted in massive renal tissue damage and deterioration of renal function in rats, which was attributed to the increased oxidative stress and the pro-inflammatory and pro-apoptotic effects of MTX. The ultrastructural observations of the effect of PRP and AD-MSCs on the kidneys of methotrexate-treated rats were observed for the first time in this study. PRP and MSC pretreatment alleviated the mediators of oxidation, inflammation, and apoptosis alongside with the morphological aberration in the kidney, fundamentally by the initiation of Nrf2/PPARγ/HO-1 signaling and NF- κB/Keap1/caspase-3 axis inhibition. This effect is considered as a hopeful solution to be tested in humans through clinical trials that aim to alleviate the renal damage produced by MTX or delay its deleterious actions. Hence, we propose the use of PRP and AD-MSCs as an adjuvant therapy to prevent and attenuate renal toxicity in patients taking MTX; however, further studies are recommended to elucidate the mode of action of the used therapeutic strategies and the long-term assessment of the transplanted cells.

## Figures and Tables

**Table 1:** Primers sequences for qRT-PCR.

Gene	Primer Sequence
Caspase-3	F5′-GGTATTGAGACAGACAGTGG-3′ R:5′-CATGGGATCTGTTTCTTTGC-3′
Keap1	F:5′-GGATGGTAACCGAACCTTCA-3′ R:5′-AAGCCCGTTGGTGAACATAG-3′
NF-κB	F:5′-CTGGCAGCTCTTCTCAAAGC-3′ R:5′-CCAGGTCATAGAGAGGCTCAA -3′
Nrf2	F:5′-CCCATTGAGGGCTGTGAT-3′ R:5′-TTGGCTGTGCTTTAGGTC-3′
PPARγ	F:5′-GGACGCTGAAGAAGAGACCTG-3′ R:5′-CCGGGTCCTGTCTGAGTATG-3′
HO-1	F:5′-GCATGTCCCAGGATTTGTCC-3′ R:5′-GGTTCTGCTTGTTTCGCTCT-3′
β-actin	F:5′-GGGAAATCGTGCGTGACATT-3′ R:5′-GCGGCAGTGGCCATCTC-3′

**Table 2:** Effect of AD-MSCs and PRP on the oxidative stress state and the levels of antioxidant enzymes in the renal tissues of methotrexate-intoxicated rats. n = 8 rats/group.

Parameters	Control	MTX	MTX + PRP	MTX + MSC
MDA	43.5 ± 4.04	90.33 ± 4.84 ^a^	55.50 ± 4.8 ^b^	49.00 ± 5.22 ^b^
SOD	279.2 ± 6.01	220.83 ± 12.88 ^a^	266.17 ± 5.95 ^b^	276.83 ± 5.23 ^b^
GSH	6.8 ± 0.36	4.05 ± 0.15 ^a^	6.25 ± 0.29 ^b^	6.43 ± 0.34 ^b^
CAT	329. ± 10.98	214.67 ± 8.24 ^a^	308.83 ± 8.45 ^b^	326.83 ± 5.34 ^bc^
TAC	90.3 ± 5.89	46.7 ± 4.89 ^a^	74.2 ± 5.78 ^ab^	83.2 ± 3.49 ^b^

Data are mean ± SD. Control; MTX: methotrexate; MTX + PRP: methotrexate + platelet rich plasma; MTX + MSC: methotrexate + mesenchymal stem cells. MDA: malondialdehyde (nmol/g tissue); SOD: superoxide dismutase (U/gm tissue); GSH: reduced glutathione (mmol/gm tissue); CAT: catalase (U/gm tissue); TAC: total antioxidant capacity (U/mg). ^a^: vs. control group; ^b^: vs. MTX group; ^c^: vs. MTX + PRP group. Significant at *p* < 0.05.

**Table 3:** Effect of AD-MSC and PRP on the levels of inflammatory markers in the renal tissues of Methotrexate –intoxicated rats. n = 8 rats/group.

Parameters	Control	MTX	MTX + PRP	MTX + MSC
IL-1β	14.9 ± 0.83	32.3 ± 1.60 ^a^	17.8 ± 0.87 ^b^	16.0 ± 0.66 ^b^
IL-6	99.2 ± 6.34	172.0 ± 5.55 ^a^	112.5 ± 10.11 ^b^	106.8 ± 10.55 ^b^
TNF-α	2364 ± 23.44	3280 ± 55.75 ^a^	2495 ± 57.11 ^b^	2424 ± 52.47 ^b^

Data are mean ± SD. Control, MTX: Methotrexate, MTX + PRP: Methotrexate +Platelet rich plasma; MTX + MSC: Methotrexate + Mesenchymal Stem cells. IL-1β: interleukin-1β (ng/g tissue); IL-6: interleukin-6 (pg/g tissue); TNF-α: tumor necrosis factor-α (pg/g tissue). ^a^: vs. control group; ^b^: vs. MTX group; ^c^: vs. MTX + PRP group. significant at *p* < 0.05.

## Data Availability

The data that support the findings of this study are available from the corresponding author upon reasonable request.

## References

[1] Lopez-Olivo M., Siddhanamatha H., Shea B., Tugwell P., A Wells G., E Suarez-Almazor M. (2014). Methotrexate for treating rheumatoid arthritis. Cochrane Database Syst. Rev..

[2] Hazlewood G.S., Barnabe C., Tomlinson G., Marshall D., Devoe D., Bombardier C. (2016). Methotrexate monotherapy and methotrexate combination therapy with traditional and biologic disease modifying antirheumatic drugs for rheumatoid ar-thritis: Abridged Cochrane systematic review and network meta-analysis. BMJ.

[3] Salliot C., van der Heijde D. (2009). Long-term safety of methotrexate monotherapy in patients with rheumatoid arthritis: A sys-tematic literature research. Ann. Rheum. Dis..

[4] Conway R., Low C., Coughlan R.J., O’Donnell M., Carey J. (2015). Risk of liver injury among methotrexate users: A meta-analysis of randomised controlled trials. Semin. Arthritis Rheum..

[5] Ibrahim A., Ahmed M., Conway R., Carey J.J. (2018). Risk of infection with methotrexate therapy in inflammatory diseases: A sys-tematic review and meta-analysis. J. Clin. Med..

[6] Ramalanjaona B., Hevroni G., Cham S., Page C., Salifu M.O., McFarlane S.I. (2020). Nephrotoxicity Associated with Low-dose Methotrexate and Outpatient Parenteral Microbial Therapy: A Case Report, Review of the Literature and Pathophysiologic Insights. Am. J. Med. Case Rep..

[7] Gilani S.T.A., Khan D.A., Khan F.A., Ahmed M. (2012). Adverse effects of low dose methotrexate in rheumatoid arthritis patients. J. Coll. Physicians Surg. Pak..

[8] Koźmiński P., Halik P.K., Chesori R., Gniazdowska E. (2020). Overview of Dual-Acting Drug Methotrexate in Different Neurological Diseases, Autoimmune Pathologies and Cancers. Int. J. Mol. Sci..

[9] Widemann B.C., Adamson P.C. (2006). Understanding and Managing Methotrexate Nephrotoxicity. Oncol..

[10] Widemann B.C., Balis F., Kim A., Boron M., Jayaprakash N., Shalabi A., O’Brien M., Eby M., Cole D.E., Murphy R.F. (2010). Glucarpidase, Leucovorin, and Thymidine for High-Dose Methotrexate-Induced Renal Dysfunction: Clinical and Pharmacologic Factors Affecting Outcome. J. Clin. Oncol..

[11] Scott H., Stefan S., Thomas K., Suzanne W. (2013). 2013 Annual Meeting of the North American Congress of Clinical Toxicology (NACCT). Clin. Toxicol..

[12] Howard S.C., McCormick J., Pui C.-H., Buddington R.K., Harvey R.D. (2016). Preventing and Managing Toxicities of High-Dose Methotrexate. Oncologist.

[13] Hassanein E.H.M., Shalkami A.S., Khalaf M.M., Mohamed W.R., Hemeida R.A.M. (2019). The impact of Keap1/Nrf2, P(38)MAPK/NF-κB and Bax/Bcl2/caspase-3 signaling pathways in the protective effects of berberine against methotrex-ate-induced nephrotoxicity. Biomed. Pharmacother..

[14] Alves R., Grimalt R. (2016). Randomized placebo-controlled, double-blind, half-head study to assess the efficacy of platelet-rich plasma on the treatment of androgenetic alopecia. Dermatol. Surg..

[15] Lynch M.D., Bashir S. (2016). Applications of platelet-rich plasma in dermatology: A critical appraisal of the literature. J. Dermatol. Treat..

[16] Alves R., Grimalt R. (2018). A Review of Platelet-Rich Plasma: History, Biology, Mechanism of Action, and Classification. Ski. Appendage Disord..

[17] Marx R.E. (2001). Platelet-Rich Plasma (PRP): What Is PRP and What Is Not PRP?. Implant. Dent..

[18] Eppley B.L., Woodell J.E., Higgins J. (2004). Platelet Quantification and Growth Factor Analysis from Platelet-Rich Plasma: Implications for Wound Healing. Plast. Reconstr. Surg..

[19] Carlson N.E., Roach R.B. (2002). Platelet-rich plasma: Clinical applications in dentistry. J. Am. Dent. Assoc..

[20] Moghadam A., Khozani T.T., Mafi A., Namavar M.R., Dehghani F. (2017). Effects of Platelet-Rich Plasma on Kidney Regeneration in Gentamicin-Induced Nephrotoxicity. J. Korean Med. Sci..

[21] Keshk W.A., Zahran S.M. (2019). Mechanistic role of cAMP and hepatocyte growth factor signaling in thioacetamide-induced nephrotoxicity: Unraveling the role of platelet rich plasma. Biomed. Pharmacother.

[22] Uccelli A., Moretta L., Pistoia V. (2008). Mesenchymal stem cells in health and disease. Nat. Rev. Immunol..

[23] Liu Z.-J., Zhuge Y., Velazquez O.C. (2009). Trafficking and differentiation of mesenchymal stem cells. J. Cell. Biochem..

[24] Ibrahim M.A., Khalifa A.M., Mohamed A.A., Galhom R.A., Korayem H.E., El-Fadeal N.M.A., Tammam A.A.-E., Khalifa M.M., Elserafy O.S., Abdel-Karim R.I. (2022). Bone-Marrow-Derived Mesenchymal Stem Cells, Their Conditioned Media, and Olive Leaf Extract Protect against Cisplatin-Induced Toxicity by Alleviating Oxidative Stress, Inflammation, and Apoptosis in Rats. Toxics.

[25] Perico L., Morigi M., Rota C., Breno M., Mele C., Noris M., Introna M., Capelli C., Longaretti L., Rottoli D. (2017). Human mesenchymal stromal cells transplanted into mice stimulate renal tubular cells and enhance mitochondrial function. Nat. Commun..

[26] Semedo P., Palasio C.G., Oliveira C.D., Feitoza C.Q., Gonçalves G.M., Cenedeze M.A., Wang P.M., Teixeira V.P., Reis M.A., Pacheco-Silva A. (2009). Early modulation of inflammation by mesenchymal stem cell after acute kidney injury. Int. Immunopharmacol..

[27] Gad A.M., Hassan W.A., Fikry E.M. (2017). Significant curative functions of the mesenchymal stem cells on methotrexate-induced kidney and liver injuries in rats. J. Biochem. Mol. Toxicol..

[28] E Weinblatt M., Maier A.L., A Fraser P., Coblyn J.S. (1998). Longterm prospective study of methotrexate in rheumatoid arthritis: Conclusion after 132 months of therapy. J. Rheumatol..

[29] Yozai K., Shikata K., Sasaki M., Tone A., Ohga S., Usui H., Okada S., Wada J., Nagase R., Ogawa D. (2005). Methotrexate Prevents Renal Injury in Experimental Diabetic Rats via Anti-Inflammatory Actions. J. Am. Soc. Nephrol..

[30] Salem N., Helmi N., Assaf N. (2018). Renoprotective Effect of Platelet-Rich Plasma on Cisplatin-Induced Nephrotoxicity in Rats. Oxidative Med. Cell. Longev..

[31] Hesami Z., Jamshidzadeh A., Ayatollahi M., Geramizadeh B., Farshad O., Vahdati A. (2014). Effect of Platelet-Rich Plasma on CCl_4_-Induced Chronic Liver Injury in Male Rats. Int. J. Hepatol..

[32] Megaloikonomos P.D., Panagopoulos G., Bami M., Igoumenou V.G., Dimopoulos L., Milonaki A., Kyriakidou M., Mitsiokapa E., Anastassopoulou J., Mavrogenis A.F. (2018). Harvesting, Isolation and Differentiation of Rat Adipose-Derived Stem Cells. Curr. Pharm. Biotechnol..

[33] Bunnell B.A., Flaat M., Gagliardi C., Patel B., Ripoll C. (2008). Adipose-derived stem cells: Isolation; expansion; differentiation. Methods.

[34] Pazzini J.M., Nardi A.B.D., Huppes R.R., Gering A.P., Ferreira M.G., Silveira C.P., Luzzi M.C., Santos R. (2016). Method to obtain platelet-rich plasma from rabbits (*Oryctolagus cuniculus*). Pesqui. Veterinária Bras..

[35] Chung B.H., Lim S.W., Doh K.C., Piao S.G., Heo S.B., Yang C.W. (2013). Human Adipose Tissue Derived Mesenchymal Stem Cells Aggravate Chronic Cyclosporin Nephrotoxicity by the Induction of Oxidative Stress. PLoS ONE.

[36] Grobe N., Leiva O., Morris M., Elased K.M. (2015). Loss of Prolyl Carboxypeptidase in Two-Kidney, One-Clip Goldblatt Hypertensive Mice. PLoS ONE.

[37] Sahin H., Yener A.U., Karaboga I., Sehitoglu M.H., Dogu T., Altinisik H.B., Altinisik U., Simsek T. (2017). Protective effect of gel form of gastric gavage applicated aloe vera on ischemia reperfusion injury in renal and lung tissue. Cell. Mol. Biol..

[38] Ohashi R., Shimizu A., Masuda Y., Kitamura H., Ishizaki M., Sugisaki Y., Yamanaka N. (2002). Peritubular Capillary Regression during the Progression of Experimental Obstructive Nephropathy. J. Am. Soc. Nephrol..

[39] Derakhshanfar A., Sadeghian M.H., Abbasabadi N., Imanian M.H. (2015). Histopathologic and biochemical study of the effect of saffron extract on gentamicin-induced nephrotoxicity in rats. Comp. Clin. Pathol..

[40] Beutler E., Duron O., Kelly B.M. (1963). Improved method for the determination of blood glutathione. J. Lab. Clin. Med..

[41] Masayasu M., Hiroshi Y. (1979). A simplified assay method of superoxide dismutase activity for clinical use. Clin. Chim. Acta.

[42] Aebi H. (1984). Catalase in vitro. Methods in Enzymology.

[43] Kei S. (1978). Serum lipid peroxide in cerebrovascular disorders determined by a new colorimetric method. Clin. Chim. Acta.

[44] Livak K.J., Schmittgen T.D. (2001). Analysis of relative gene expression data using real-time quantitative PCR and the 2(-Delta Delta C(T)) Method. Methods.

[45] Andankar P., Shah K., Patki V. (2018). A review of drug-induced renal injury. J. Pediatr. Crit. Care.

[46] Asci H., Ozmen O., Ellidag H.Y., Aydin B., Bas E., Yilmaz N. (2017). The impact of gallic acid on the methotrexate-induced kidney damage in rats. J. Food Drug Anal..

[47] Heidari R., Ahmadi A., Mohammadi H., Ommati M.M., Azarpira N., Niknahad H. (2018). Mitochondrial dysfunction and oxida-tive stress are involved in the mechanism of methotrexate-induced renal injury and electrolytes imbalance. Biomed. Pharmacother..

[48] Sharifi-Rad M., Anil Kumar N.V., Zucca P., Varoni E.M., Dini L., Panzarini E., Rajkovic J., Tsouh Fokou P.V., Azzini E., Peluso I. (2020). Lifestyle, Oxidative Stress, and Antioxidants: Back and Forth in the Pathophysiology of Chronic Diseases. Front. Physiol..

[49] Ogrodnik M., Salmonowicz H., Gladyshev V.N. (2019). Integrating cellular senescence with the concept of damage accumulation in aging: Relevance for clearance of senescent cells. Aging Cell.

[50] Lin S., Lin W., Liao C., Zhou T. (2020). Nephroprotective Effect of Mesenchymal Stem Cell-Based Therapy of Kidney Disease In-duced by Toxicants. Stem Cells Int..

[51] Chen Q.M., Maltagliati A.J. (2018). Nrf2 at the heart of oxidative stress and cardiac protection. Physiol. Genom..

[52] Shen Y., Liu X., Shi J., Wu X. (2019). Involvement of Nrf2 in myocardial ischemia and reperfusion injury. Int. J. Biol. Macromol..

[53] Bellezza I., Giambanco I., Minelli A., Donato R. (2018). Nrf2-Keap1 signaling in oxidative and reductive stress. Biochim. Biophys. Acta (BBA)-Mol. Cell Res..

[54] Yamamoto M., Kensler T.W., Motohashi H. (2018). The KEAP1-NRF2 System: A Thiol-Based Sensor-Effector Apparatus for Main-taining Redox Homeostasis. Physiol. Rev..

[55] Lian F.Z., Cheng P., Ruan C.S., Ling X.X., Wang X.Y., Pan M., Chen M.L., Shen A.Z., Gao S. (2019). Xin-Ji-Er-Kang ameliorates kidney injury following myocardial infarction by inhibiting oxidative stress via Nrf2/HO-1 pathway in rats. Biomed. Pharmacother..

[56] Ding M., Li M., Zhang E.M., Yang H.L. (2020). FULLEROL alleviates myocardial ischemia-reperfusion injury by reducing in-flammation and oxidative stress in cardiomyocytes via activating the Nrf2/HO-1 signaling pathway. Eur. Rev. Med. Pharmacol. Sci..

[57] Cheng J., Zhang M., Cheng S., Li F., Zhang B., Sun X., Hu H., Chen L., Zhao Z., Hu H. (2021). Low-dose alcohol ameliorated high fat diet-induced anxiety-related behavior *via* enhancing adiponectin expression and activating the Nrf2 pathway. Food Funct..

[58] Qiao H., Sai X., Gai L., Huang G., Chen X., Tu X., Ding Z. (2014). Association between heme oxygenase 1 gene promoter poly-morphisms and susceptibility to coronary artery disease: A HuGE review and meta-analysis. Am. J. Epidemiology.

[59] Lu M.-C., Ji J.-A., Jiang Y.-L., Chen Z.-Y., Yuan Z.-W., You Q.-D., Jiang Z.-Y. (2016). An inhibitor of the Keap1-Nrf2 protein-protein interaction protects NCM460 colonic cells and alleviates experimental colitis. Sci. Rep..

[60] Li A., Zhang X., Luo Q. (2021). Neohesperidin alleviated pathological damage and immunological imbalance in rat myocardial ischemia-reperfusion injury via inactivation of JNK and NF-κB p65. Biosci. Biotechnol. Biochem..

[61] Andia I., Abate M. (2018). Platelet-rich plasma: Combinational treatment modalities for musculoskeletal conditions. Front. Med..

[62] Nguyen R.T., Borg-Stein J., McInnis K. (2011). Applications of platelet-rich plasma in musculoskeletal and sports medicine: An evi-dence-based approach. PM R J. Inj. Funct. Rehabil..

[63] Qureshi A.H., Chaoji V., Maiguel D., Faridi M.H., Barth C.J., Salem S.M., Singhal M., Stoub D., Krastins B., Ogihara M. (2009). Proteomic and Phospho-Proteomic Profile of Human Platelets in Basal, Resting State: Insights into Integrin Signaling. PLoS ONE.

[64] Alsousou J., Ali A., Willett K., Harrison P. (2012). The role of platelet-rich plasma in tissue regeneration. Platelets.

[65] Foster T.E., Puskas B.L., Mandelbaum B.R., Gerhardt M.B., Rodeo S.A. (2009). Platelet-rich plasma: From basic science to clinical applications. Am. J. Sport. Med..

[66] Madrazo J.A., Kelly D.P. (2008). The PPAR trio: Regulators of myocardial energy metabolism in health and disease. J. Mol. Cell. Cardiol..

[67] Ma Y., Shi M., Wang Y., Liu J. (2020). PPAR*γ* and Its Agonists in Chronic Kidney Disease. Int. J. Nephrol..

[68] Borrione P., Gianfrancesco A.D., Pereira M.T., Pigozzi F. (2010). Platelet-rich plasma in muscle healing. Am. J. Phys. Med. Rehabil..

[69] Lubkowska A., Dolegowska B., Banfi G. (2013). Growth factor content in PRP and their applicability in medicine. J. Biol. Regul. Homeost. Agents.

[70] El-Sharkawy H., Kantarci A., Deady J., Hasturk H., Liu H., Alshahat M., Van Dyke T.E. (2007). Platelet-Rich Plasma: Growth Factors and Pro- and Anti-Inflammatory Properties. J. Periodontol..

[71] Moussa M., Lajeunesse D., Hilal G., El Atat O., Haykal G., Serhal R., Chalhoub A., Khalil C., Alaaeddine N. (2017). Platelet rich plasma (PRP) induces chondroprotection via increasing autophagy, anti-inflammatory markers, and decreasing apoptosis in human osteoarthritic cartilage. Exp. Cell Res..

[72] Sadeghinia A., Davaran S., Salehi R., Jamalpoor Z. (2019). Nano-hydroxy apatite/chitosan/gelatin scaffolds enriched by a combi-nation of platelet-rich plasma and fibrin glue enhance proliferation and differentiation of seeded human dental pulp stem cells. Biomed. Pharmacother..

[73] Sundman E.A., Cole B.J., Karas V., Della Valle C., Tetreault M.W., Mohammed H.O., Fortier L.A. (2014). The Anti-inflammatory and Matrix Restorative Mechanisms of Platelet-Rich Plasma in Osteoarthritis. Am. J. Sports Med..

[74] El-Twab S.M.A., Hussein O.E., Hozayen W.G., Bin-Jumah M., Mahmoud A. (2019). MChicoric acid prevents methotrexate-induced kidney injury by suppressing NF-κB/NLRP3 inflammasome activation and up-regulating Nrf2/ARE/HO-1 signaling. Inflamm. Res. Off. J. Eur. Histamine Res. Soc..

[75] Mahmoud A.M., Hussein O.E., Hozayen W.G., Bin-Jumah M., El-Twab S.M.A. (2020). Ferulic acid prevents oxidative stress, in-flammation, and liver injury via upregulation of Nrf2/HO-1 signaling in methotrexate-induced rats. Environ. Sci. Pollut. Res. Int..

[76] Rahnavard M., Hassanpour M., Ahmadi M., Heidarzadeh M., Amini H., Javanmard M.Z., Nouri M., Rahbarghazi R., Safaie N. (2019). Curcumin ameliorated myocardial infarction by inhibition of cardiotoxicity in the rat model. J. Cell. Biochem..

[77] Zhang W., Li Y., Ge Z. (2017). Cardiaprotective effect of crocetin by attenuating apoptosis in isoproterenol induced myocardial infarction rat model. Biomed. Pharmacother..

[78] Zhou M., Liu X., Li Z., Huang Q., Li F., Li C.-Y. (2018). Caspase-3 regulates the migration, invasion and metastasis of colon cancer cells. Int. J. Cancer.

[79] Akhigbe R., Ajayi A. (2020). Testicular toxicity following chronic codeine administration is via oxidative DNA damage and up-regulation of NO/TNF-α and caspase 3 activities. PLoS ONE.

[80] Yang C.-C., Chen Y.-T., Wallace C.G., Chen K.-H., Cheng B.-C., Sung P.-H., Li Y.-C., Ko S.-F., Chang H.-W., Yip H.-K. (2019). Early administration of empagliflozin preserved heart function in cardiorenal syndrome in rat. Biomed. Pharmacother..

[81] Zhang F.-X., Yuan Y.-L., Cui S.-S., Li M., Tan X., Qiu Z.-C., Li R.-M. (2021). Dissection of the potential pharmacological function of neohesperidin dihydrochalcone–a food additive–by in vivo substances profiling and network pharmacology. Food Funct..

[82] Seo H.Y., Lee S.H., Lee J.H., Hwang J.S., Kim M.K., Jang B.K. (2020). Kahweol activates the Nrf2/HO-1 pathway by decreasing Keap1 expression independently of p62 and autophagy pathways. PLoS ONE.

[83] Hacioglu C., Kar F., Kacar S., Sahinturk V., Kanbak G. (2021). Bexarotene inhibits cell proliferation by inducing oxidative stress, DNA damage and apoptosis via PPARγ/NF-κB signaling pathway in C6 glioma cells. Med. Oncol..

[84] Gendy A.M., Amin M.M., Al-Mokaddem A.K., Ellah M.F.A. (2021). Cilostazol mitigates mesenteric ischemia/reperfusion-induced lung lesion: Contribution of PPAR-γ, NF-κB, and STAT3 crosstalk. Life Sci..

[85] Saito Y., Kuse Y., Inoue Y., Nakamura S., Hara H., Shimazawa M. (2018). Transient acceleration of autophagic degradation by pharmacological Nrf2 activation is important for retinal pigment epithelium cell survival. Redox Biol..

[86] Hussein O.E., Hozayen W.G., Bin-Jumah M.N., Germoush M.O., El-Twab S.M.A., Mahmoud A.M. (2020). Chicoric acid prevents methotrexate hepatotoxicity via attenuation of oxidative stress and inflammation and up-regulation of PPARγ and Nrf2/HO-1 signaling. Environ. Sci. Pollut. Res. Int..

[87] Li C., Zhang J., Xue M., Li X., Han F., Liu X., Xu L., Lu Y., Cheng Y., Li T. (2019). SGLT2 inhibition with empagliflozin attenuates myocardial oxidative stress and fibrosis in diabetic mice heart. Cardiovasc. Diabetol..

[88] Dominici M., Le Blanc K., Mueller I., Slaper-Cortenbach I., Marini F.C., Krause D.S., Deans R.J., Keating A., Prockop D.J., Horwitz E.M. (2006). Minimal criteria for defining multipotent mesenchymal stromal cells. The International Society for Cellular Therapy position statement. Cytotherapy.

[89] Czapla J., Matuszczak S., Kulik K., Wiśniewska E., Pilny E., Jarosz-Biej M., Smolarczyk R., Sirek T., Zembala M.O., Zembala M. (2019). The effect of culture media on large-scale expansion and characteristic of adipose tis-sue-derived mesenchymal stromal cells. Stem Cell Res. Ther..

[90] El-Agawy M.S.E.-D., Badawy A.M.M., Rabei M.R., Elshaer M.M.A., El Nashar E.M., Alghamdi M.A., Alshehri M.A., Elsayed H.R.H. (2022). Methotrexate-Induced Alteration of Renal Aquaporins 1 and 2, Oxidative Stress and Tubular Apoptosis Can Be Attenuated by Omega-3 Fatty Acids Supplementation. Int. J. Mol. Sci..

[91] Dabak D.O., Kocaman N. (2015). Effects of silymarin on methotrexate-induced nephrotoxicity in rats. Ren. Fail..

[92] Elsawy H., Alzahrani A.M., Alfwuaires M., Abdel-Moneim A.M., Khalil M. (2021). Nephroprotective effect of naringin in metho-trexate induced renal toxicity in male rats. Biomed. Pharmacother..

[93] Wei X., Wu Y., Tang H., Wang B., Wang Y., Sun W., Asenso J., Xiao F., Wang C. (2021). CP-25 ameliorates methotrexate induced nephrotoxicity via improving renal apoptosis and methotrexate excretion. J. Pharmacol. Sci..

[94] Babiak R.M.V., Campello A.P., Carnieri E.G., Oliveira M.B.M. (1998). Methotrexate: Pentose cycle and oxidative stress. Cell Biochem. Funct. Cell. Biochem. Its Modul. Act. Agents Dis..

[95] Vardi N., Parlakpinar H., Ates B., Cetin A., Otlu A. (2013). The protective effects of Prunus armeniaca L (apricot) against metho-trexate-induced oxidative damage and apoptosis in rat kidney. J. Physiol. Biochem..

[96] Fu Y., Karbaat L., Wu L., Leijten J., Both S.K., Karperien M. (2017). Trophic Effects of Mesenchymal Stem Cells in Tissue Regeneration. Tissue Eng. Part B Rev..

[97] Ankrum J., Karp J.M. (2010). Mesenchymal stem cell therapy: Two steps forward, one step back. Trends Mol. Med..

[98] Ahmed S.M., Fouad F.E. (2019). Possible protective effect of platelet-rich plasma on a model of cisplatin-induced nephrotoxicity in rats: A light and transmission electron microscopic study. J. Cell. Physiol..

[99] Afifi N.M., Reyad O.N. (2013). Role of mesenchymal stem cell therapy in restoring ovarian function in a rat model of chemothera-py-induced ovarian failure: A histological and immunohistochemical study. Egypt. J. Histol..

[100] Choi M.R., Kim H.Y., Park J.-Y., Lee T.Y., Baik C.S., Chai Y.G., Jung K.H., Park K.S., Roh W., Kim K.S. (2010). Selection of optimal passage of bone marrow-derived mesenchymal stem cells for stem cell therapy in patients with amyotrophic lateral sclerosis. Neurosci. Lett..

[101] Ebrahimi R., Sepand M.R., Seyednejad S.A., Omidi A., Akbariani M., Gholami M., Sabzevari O. (2019). Ellagic acid reduces methotrexate-induced apoptosis and mitochondrial dysfunction via up-regulating Nrf2 expression and inhibiting the IĸBα/NFĸB in rats. DARU J. Pharm. Sci..

[102] Morsy M.A., Abdel-Aziz A.M., Abdel-Hafez S.M.N., Venugopala K.N., Nair A.B., Abdel-Gaber S.A. (2020). The Possible Contri-bution of P-Glycoprotein in the Protective Effect of Paeonol against Methotrexate-Induced Testicular Injury in Rats. Pharmaceuticals.

[103] Abbas M., El-Sherbeney S., Ahmed R. (2016). The Possible Ameliorative effect of Zafirlukast on Renal Toxicity Induced by Methotrexate in Experimental Animals. Ain Shams J. Forensic Med. Clin. Toxicol..

